# Effect of Computer-Based Substance Use Screening and Brief Behavioral Counseling vs Usual Care for Youths in Pediatric Primary Care: A Pilot Randomized Clinical Trial

**DOI:** 10.1001/jamanetworkopen.2019.6258

**Published:** 2019-06-21

**Authors:** John R. Knight, Lon Sherritt, Erin Bray Gibson, Jordan A. Levinson, Laura K. Grubb, Ronald C. Samuels, Thomas Silva, Louis Vernacchio, Wendy Wornham, Sion Kim Harris

**Affiliations:** 1 Department of Psychiatry, Boston Children’s Hospital, Boston, Massachusetts; 2 Department of Medicine, Boston Children’s Hospital, Boston, Massachusetts; 3 The Center for Adolescent Substance Use and Addiction Research, Boston Children’s Hospital, Boston, Massachusetts; 4 Department of Pediatrics, Harvard Medical School, Boston, Massachusetts; 5 Division of Adolescent and Young Adult Medicine, Boston Children’s Hospital, Boston, Massachusetts; 6 Department of Pediatrics, Floating Hospital for Children at Tufts Medical Center, Boston, Massachusetts; 7 Division of General Academic Pediatrics, Boston Children’s Hospital, Boston, Massachusetts; 8 East Boston Neighborhood Health Center, Boston, Massachusetts; 9 Longwood Pediatrics, Boston, Massachusetts; 10 Lexington Pediatrics, Lexington, Massachusetts

## Abstract

**Importance:**

Annual preventive health visits provide an opportunity to screen youths for unhealthy substance use and intervene before serious harm results.

**Objectives:**

To assess the feasibility and acceptability and estimate the efficacy of a primary care computer-facilitated screening and practitioner-delivered brief intervention (CSBI) system compared with usual care (UC) for youth substance use and associated risk of riding with an impaired driver.

**Design, Setting, and Participants:**

An intent-to-treat pilot randomized clinical trial compared CSBI with UC among 965 youths aged 12 to 18 years at 5 pediatric primary care offices and 54 practitioners. Patients were randomized to CSBI (n = 628) or usual care (n = 243) groups within practitioner with 12 months of follow-up. Data were collected from February 1, 2015, to December 31, 2017. Data analysis was performed January 1, 2018, to March 30, 2019.

**Interventions:**

Patients self-administered a computer-facilitated substance use screening questionnaire before their annual preventive health visits. Immediately after completing the screening, they received their score and level of risk and viewed 10 pages of scientific information and true-life vignettes illustrating health risks associated with substance use. Trained practitioners received the screening results, patients’ risk levels, talking points designed to prompt brief counseling, and recommended follow-up plans.

**Main Outcomes and Measures:**

Feasibility and acceptability were assessed using adolescents’ postvisit ratings. Days of alcohol use, cannabis use, and heavy episodic drinking were assessed at baseline and 3-, 6-, 9-, and 12-month follow-ups using Timeline Followback, and riding in the past 3 months with a driver who was impaired by use of alcohol or other drugs was assessed using 2 self-report items. The primary outcome was the intervention effect among at-risk youths who reported using alcohol or other drugs in the past 12 months or riding with an impaired driver in the past 3 months at baseline. The secondary outcome was the prevention effect among those with no prior use or risk.

**Results:**

Among 871 youths screened, 869 completed the baseline assessment; 211 of the 869 reported alcohol or cannabis use in the past 12 months at baseline (mean [SD] age, 16.4 [1.3] years; 114 [54.1%] female; 105 [49.8%] non-Hispanic white). Of the 211 youths, 148 (70.1%) were assigned to the CSBI group and 63 (29.9%) were assigned to the UC group. Among youths in the CSBI group, 105 (70.9%) reported receiving counseling about alcohol, 122 (82.4%) reported receiving counseling about cannabis, and 129 (87.2%) reported receiving counseling about not riding with an impaired driver. Adjusted hazard ratios for time to first postvisit use of alcohol or other drugs for CSBI vs UC were as follows: alcohol use, 0.69 (95% CI, 0.47-1.02); heavy episodic drinking, 0.66 (95% CI, 0.40-1.10); and cannabis use, 0.62 (95% CI, 0.41-0.94). At 12-month follow-ups among 99 youths who reported having ridden in the past 3 months at baseline with an impaired driver (64 in the CSBI group; 35 in the UC group), adjusted relative risk ratio of riding in the past 3 months with an impaired driver for CSBI vs UC groups was 0.58 (95% CI, 0.37-0.91). No intervention effect was observed among youths who reported no prior use of alcohol or other drugs (n = 658) or not having ridden with an impaired driver (n = 769) at baseline.

**Conclusions and Relevance:**

The CSBI system is a feasible and acceptable option for screening youths in primary care practice for use of alcohol and other drugs and for risk of riding with an impaired driver, and the estimated efficacy in this sample warrants further testing in larger samples.

**Trial Registration:**

ClinicalTrials.gov identifier: NCT00227877

## Key Points

**Question:**

Is a computer-facilitated system for youth substance use screening and brief intervention (CSBI) feasible and acceptable compared with usual care (UC) in primary care?

**Findings:**

In this pilot randomized clinical trial, among 106 youths who reported cannabis use in the past 12 months at baseline, the CSBI group reported longer time to first postvisit cannabis use compared with the UC group. Among 99 youths who reported riding with an impaired driver in the past 3 months at baseline, the CSBI group reported a lower rate of having ridden with an impaired driver in the past 3 months compared with the UC group.

**Meaning:**

The CSBI system is feasible and acceptable in clinical practice and should be further tested in larger samples.

## Teaser

This pilot randomized clinical trial assesses the feasibility and acceptability of a computer-facilitated screening questionnaire for substance use and practitioner-delivered brief intervention vs usual care among youths at primary care clinics.

## Introduction

By their senior year in high school, more than 60% of students have begun to drink alcohol, 45% of students have been drunk, and 45% of students have used marijuana or hashish.^1^ A 2017 survey^2^ reported that among students in grades 9 through 12, 30% reported drinking, 13.5% reported heavy episodic drinking (HED; defined as ≥4 drinks in a row for girls and ≥5 drinks in a row for boys^3^), 19.8% reported using marijuana, and 16.5% reported riding in a car with a driver who had been drinking alcohol, all during the 30 days prior to the survey. Use of alcohol and drugs is strongly linked to the leading causes of death among adolescents, including motor vehicle crashes and other unintentional injuries, homicides, suicides, and an array of other serious health risks and problems.^2^

More than 80% of children and adolescents aged 6 to 17 years have a medical office visit annually,^4^ and pediatricians are a consistent and trusted presence in their lives,^5^ making primary care offices a logical venue for screening and early intervention. The American Academy of Pediatrics and the National Institute on Alcohol Abuse and Alcoholism both recommend annual screening and behavioral counseling for alcohol use as a routine part of adolescent care.^3,6^ However, a 2013 study by Hingson et al^7^ reported that only about half of adolescents who had a visit with a physician in the past year reported being asked about alcohol use. Practitioner-reported barriers to screening include lack of time, insufficient training in how to manage positive screens, and unfamiliarity with validated screening tools.^8^ Therefore, even performed screenings can be of low quality, resulting in missed opportunities for early identification and intervention.^9,10,11^ Primary care screening and behavioral counseling for hazardous drinking are widely recommended for adults.^12,13^ However, in its most recent evidence reviews, the US Preventive Services Task Force found insufficient evidence to recommend for or against primary care screening and brief counseling of adolescents for either alcohol or drug use.^14,15^

To address this evidence gap, we developed a computer-facilitated screening and brief intervention (CSBI) office system, including a self-administered screening questionnaire based on the CRAFFT (car, relax, alone, forget, family or friends, trouble) screening tool,^16^ immediate personalized feedback and psychoeducation, and point-of-care decision support for practitioners. The aims of our study were to assess the acceptability of the CSBI system in primary care and to generate randomized clinical trial–based estimates of effect size for youth substance use and the safety risk of riding with an impaired driver, defined by the judgment of the participating youth. Of primary interest was the intervention effect among at-risk youths, ie, those who reported prior substance use or riding risk. Secondarily, we examined the prevention effect among youths with no reported prior use of alcohol or other drugs or riding risk.

## Methods

### Study Design and Participants

This study follows the Consolidated Standards of Reporting Trials (CONSORT) reporting guideline. We conducted a pilot randomized clinical trial from February 1, 2015, to December 31, 2017, as part of a parent study^17^ whose primary purpose was to assess the psychometric properties of the National Institute on Alcohol Abuse and Alcoholism recommended screening for children and adolescents (the original study protocol and statistical analysis plan are presented in Supplement 1).^3^ We conducted the study in 3 community practices and 2 hospital-based practices in the greater Boston, Massachusetts, area. Data analysis was performed January 1, 2018, to March 20, 2019. The institutional review boards of all participating institutions approved the study protocol. All participating practitioners provided written informed consent. We obtained written informed assent from patients aged 12 to 17 years or written informed consent from patients aged 18 years. We received a waiver of written parental or guardian consent from the Boston Children’s Hospital and the Tufts Health Sciences institutional review boards, as requiring such consent in our 2008 study^18^ was associated with diminished recruitment and significant self-selection bias.

#### Practitioners

Practice leaders at each site sent emails inviting 80 practitioners to participate. Practitioners who worked fewer than 4 sessions per week or had fewer than 8 adolescent patients per week were excluded. All included practitioners attended a 1-hour orientation session that comprised a demonstration of the tablet computer program, a review of practitioner reports for various categories of risk, the study safety protocol, and a 20-minute video showing examples of brief counseling based on suggested talking points. They also completed a 1-hour online training session with video examples of practitioner counseling and attended a 1-hour motivational interviewing skills development training session. They received 3 American Medical Association Physician’s Recognition Award Category 1 continuing medical education credits for their participation and a $10 gift card after completing the end-of-study evaluation form.

#### Patients

**Figure 1.**
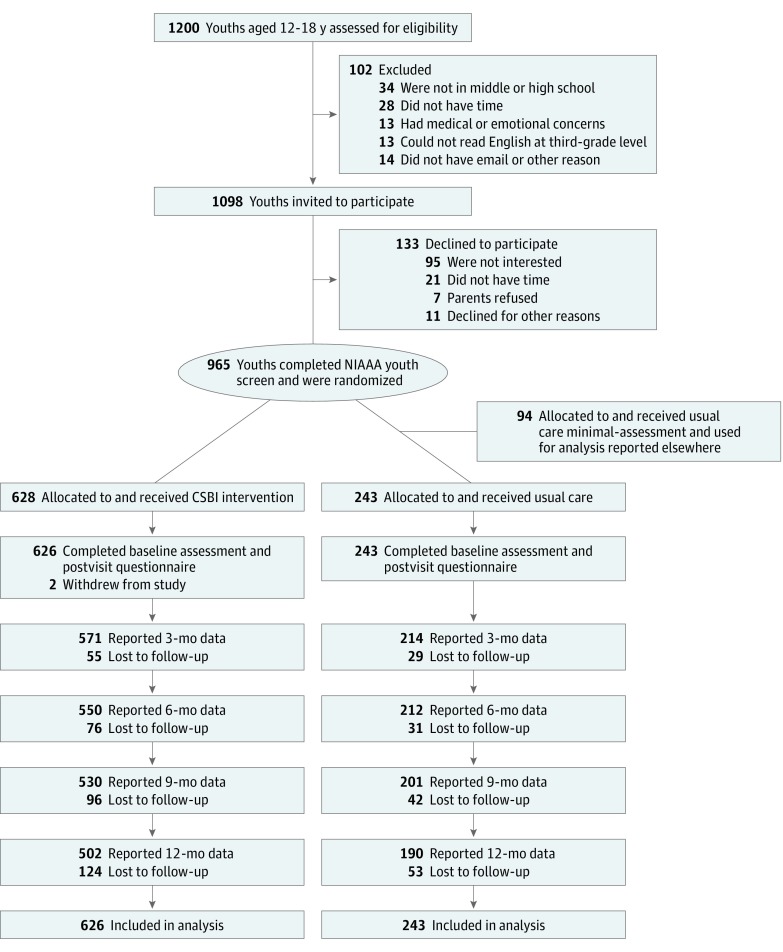
CONSORT Flowchart for Youth Recruitment and Retention CSBI indicates computer-facilitated screening and brief intervention; NIAAA, National Institute on Alcohol Abuse and Alcoholism.

We consecutively screened patients aged 12 to 18 years who presented for annual preventive health visits (Figure 1). Exclusion criteria included practitioner determination of medical or emotional instability at baseline, inability to read English at a third-grade level, or unavailability for follow-up visits during the next 12 months. Whenever possible, research associates first mailed a letter to families describing the study and providing opt-out instructions and followed up with a telephone call to patients to explain the study purpose, procedures, and confidentiality protections and to instruct interested patients to arrive 30 minutes before their appointment. On arrival, research associates privately obtained written informed assent from patients aged 12 to 17 years or written informed consent from patients 18 years or older. Parents or guardians were made aware of the study by reading the letter mailed by research associates, taking telephone messages, or accompanying their child to the clinic visit.

Patients who assented or consented were randomized within practitioner to 1 of 3 arms in a 65% CSBI, 25% usual care (UC), and 10% minimal-assessment UC allocation ratio. The minimal-assessment UC group, which completed the 12-month follow-up only, was used to address an aim in the psychometric study and was not analyzed in this study. We chose to allocate more patients to the CSBI group so that practitioners would have more experience delivering the intervention. On completion of the baseline assessment, the computerized study management system randomized patients within practitioner based on an adaptive, biased-coin minimization scheme,^19,20^ in which we minimized imbalance on the following factors, ranked by priority: grade group (middle vs high school), sex, and any drug use. Because practitioners had to deliver the intervention, they were not blinded to patients assigned to the intervention effect arm. However, for patients in the UC arm, practitioners were not informed that the patient was participating in the study.

Two patients in the CSBI group subsequently withdrew from the study. Our recruitment sample size was determined to address the parent study’s aims, not for demonstrating efficacy of the CSBI intervention.

#### Intervention

The tablet-based CSBI system started by explaining that responses to the screening questions would be kept confidential unless the practitioner identified an immediate risk to safety. In addition, practitioners asked parents or guardians to leave the room for a portion of the visit to allow a private discussion, which should be a routine part of pediatric practice for adolescents. The patient then self-administered the screening questionnaire that assessed the number of days of alcohol, cannabis, and other drug use in the past 12 months and the 6 CRAFFT questions.^16^ The tablet then immediately displayed to patients their CRAFFT score and level of risk, followed by 10 interactive pages of scientific information and true-life vignettes illustrating the health risks of substance use. This took on average 4 minutes to complete in a private location before the visit. Practitioners then logged into the tablet to see the patient’s screen results, risk level, talking points designed to prompt a 2- to 5-minute conversation using motivational interviewing strategies, and the recommended follow-up plan. Practitioners gave a printed Contract for Life^21^ to all patients (and parents or guardians, if present) as a prevention strategy for high- and low-risk patients. The Contract for Life asks youths to agree never to drive after substance use or accept a ride from a substance-impaired driver and instead to call a parent, guardian, or other trusted adult for a safe ride home. Parents or guardians agree to provide safe and sober transportation home and postpone discussion until the following day. Practitioners instructed patients to discuss the Contract for Life with their parent(s) or guardian(s) and to follow up if additional discussion was needed. Practitioners also gave all patients a flyer for a 20-minute family-centered online educational program, Teen-Safe,^22^ on preventing adolescent substance use.

The content of psychoeducational pages of CSBI was developed and tested in previous research during an iterative process of focus group discussion followed by multiple cycles of user testing with feedback and revision, and the content found favorable results in a 2012 quasi-experimental trial.^23^ In the same trial, the Contract for Life had similar, favorable results for reducing risk of riding with an impaired driver 3 months after the intervention.^24^ The content for Teen-Safe^22^ was developed by 2 of us (J.R.K. and S.K.H.) for educational presentations to parents and guardians of high school students. The parents and guardians rated Teen-Safe highly, but it has not been otherwise tested.

#### Usual Care

Patients in the UC group self-administered the computer screening but did not receive any other CSBI components. However, all practitioners received training in the CSBI, which may have affected their delivery of substance use–related counseling with patients in the UC group.

### Study Assessments

Patients completed a 15-minute baseline assessment battery prior to seeing their practitioner that included sociodemographic questions; Timeline Followback^25,26^ (TLFB) for the previous 12 months, which uses a calendar to aid recall of the frequency of use of each substance and scales to assess perceived substance use by peers, siblings, and parents derived from the Personal Experience Inventory^27,28^; and riding risk in the past 3 months assessed by 2 items (“In the past 3 months, how many times did you ride with a driver who had been drinking [using marijuana or any other drug]?” followed by 4 response options [not at all, once, twice, and 3 or more times]). To evaluate subjective reactions to the CSBI psychoeducational content, we asked CSBI participants to rate how much they felt the information was “useful,” “exaggerated,” “convincing,” and “irritating” on a scale from 1, indicating not at all, to 7, extremely. Immediately after the medical visit, patients reported on the number of previous visits they had had with the practitioner, receipt of screening and counseling, and ratings of the current visit and responded to open-ended questions about their experience during the visit and whether anything was confusing (“Was there anything you found confusing?”) or uncomfortable (“Was there anything that made you uncomfortable?”).

Trained research associates, blinded to patient screening results, administered the TLFB at baseline and reminded participants to complete it by computer self-administration at each 3-, 6-, 9-, and 12-month follow-up. To maximize data completeness for patients who missed any surveys, the TLFB asked patients to enter data for all months since their last completed survey. Participants received up to $60 in merchandise gift cards based on the number of completed assessments.

Our primary measures of feasibility and acceptability among youths were immediate postvisit reports of receiving counseling about avoiding alcohol and drug use, ratings of counseling quality, receipt of the Contract for Life, and, among those reporting receipt, whether they had discussed it with their parents or guardians by the 3-month follow-up. Among those who reported having used alcohol in the past 12 months at baseline, our primary alcohol use outcome measures were the TLFB-derived time to first postvisit alcohol use and time to first postvisit HED episode. For drug use, we examined time to first postvisit cannabis use among those who reported having used cannabis in the past 12 months at baseline. The prevalence of other drug use at baseline was too low (<1%) for analysis. Finally, we examined the intervention effect on rates of self-reported riding risk at each follow-up. We could not analyze driving risk owing to insufficient numbers (<1%).

### Statistical Analysis

We analyzed intent-to-treat groups, with the intervention effect cohort including youths assigned to CSBI regardless of whether they reported receiving practitioner counseling. We conducted analyses using SUDAAN statistical software version 11.0 (RTI International), with practitioner as the nest variable to account for correlated error arising from our cluster-sampling design. To assess baseline group equivalence, we used χ^2^ tests for categorical variables and *t* tests for continuous variables. We dichotomized race (non-Hispanic white vs other), parents in home (2 vs other), and parent education level (college graduate vs other) to ensure adequate cell sizes. We evaluated CSBI receipt and feasibility and acceptability variables using multivariable logistic regression modeling with generalized estimating equations to compute adjusted relative risk ratios (ARRRs) for CSBI compared with UC, while controlling for baseline group differences. *P* values were 2-tailed and considered statistically significant at less than .05.

We stratified analyses by baseline substance use in the past 12 months to separately assess intervention effects among youths who reported substance use and prevention effects among youths who reported no substance use. To examine the effect of the CSBI intervention on the number of days to first postvisit alcohol use, HED episode, and cannabis use, we used Cox proportional hazards modeling to compute adjusted hazard ratios that controlled for any baseline group differences. Time data for participants with at least 1 follow-up were included in analyses; those with no substance use during follow-up were censored.

To evaluate riding risk in the past 3 months, we combined any riding with a driver who had been drinking alcohol and any riding with a driver who had been using drugs into a single variable and used multivariable logistic regression with generalized estimating equations to compute ARRRs at each follow-up. These analyses were stratified by report of riding risk in the past 3 months at baseline rather than use of alcohol or other drugs in the past 12 months. Because of a technical problem with riding risk data at 3-month follow-up, we present only 6-, 9-, and 12-month outcomes.

## Results

### Baseline Sample Characteristics

Of the 80 invited practitioners, 54 (68%) agreed to participate and completed training, including 39 pediatricians (49%) and 15 nurse practitioners (19%). Of 1200 patients initially screened, we invited 1098 (91.5%) to participate, and 965 patients (87.9%) assented or consented and were randomized to 1 of the study arms: CSBI (n = 628), UC (n = 243), or minimal-assessment UC arm (n = 94). The minimal-assessment UC arm was used only in psychometric analyses and not included in outcome analyses. Two patients in the CSBI group withdrew from the study prior to completing the baseline assessment. Patients who reported any substance use or riding risk at baseline composed the intervention effect cohort, and patients who reported no substance use or riding risk composed the prevention effect cohort.

The intervention effect cohort included 211 patients (24.3%) who reported any use of alcohol or cannabis in the past 12 months at baseline (alcohol, 192 [22.1%]; cannabis, 106 [12.2%]). Mean (SD) age was 16.4 (1.3) years, 114 (54.1%) were female, 105 (49.8%) were non-Hispanic white, and 144 (70.9%) had a college-educated parent (Table 1). Among this intervention effect cohort, most patients (178 [84.4%]) reported seeing a pediatrician, 149 patients (70.6%) saw a female practitioner, and more than half (123 [58.3%]) reported 6 or more previous visits with the practitioner. Nearly all (192 [91.4%]) reported using alcohol in the past 12 months, with a median (interquartile range [IQR]) of 3 (2-6) drinking days, and 70 patients (33.2%) reported at least 1 HED episode. Of the 211 patients who reported any use of alcohol or other drugs, 106 (50.2%) reported cannabis use in the past 12 months, with a median (IQR) of 3.5 (2-15) days of use. Fifty-nine patients (28.1%) met criteria for high risk of a substance use disorder (CRAFFT score ≥2),^29^ and 56 patients (26.5%) reported having ridden in a car in the past 3 months with a driver who had used alcohol or other drugs at baseline. Most patients (188 [89.1%]) reported having close friends who used alcohol or drugs, and 82 patients (42.7%) had siblings who used alcohol or drugs. The CSBI and UC groups did not differ significantly on any baseline variable, except number of days of drinking during the past 12 months; we controlled for this variable in all further analyses. All patients completed the postvisit questionnaire before leaving the office, 717 patients (82.5%) completed at least 1 follow-up (CSBI, 529 of 626 patients [84.5%]; UC, 189 of 243 patients [77.8%]; *P* = .15), and 692 patients (79.6%) completed the 12-month follow-up (CSBI, 502 of 626 patients [80.2%]; UC, 190 of 243 patients [78.2%]; *P* = .09) (Figure 1).

**Table 1.** Baseline Characteristics of Youths Who Reported Use of Alcohol or Other Drugs in the Previous 12 Months at Baseline

Characteristic	No. (%)			Test Statistic^a^	*P* Value
	Total (n = 211)	CSBI Group (n = 148)	UC Group (n = 63)		
Age, mean (SD), y	16.4 (1.3)	16.3 (1.3)	16.5 (1.3)	.76	.45
In grades 9-12	201 (95.3)	141 (95.3)	60 (95.2)	0	.99
Female sex	114 (54.0)	80 (54.1)	34 (54.0)	0	.99
Race/ethnicity					.34
Non-Hispanic white	105 (49.8)	69 (46.6)	36 (57.1)	.10	NA
Hispanic	55 (26.1)	40 (27.0)	15 (23.8)		
Other or multirace	51 (24.2)	39 (26.4)	12 (19.0)		
2 Parents at home	144 (68.2)	103 (69.6)	41 (65.1)	.04	.52
College-graduate parent(s) or guardian(s)^b^	144 (70.9)	100 (69.4)	44 (74.6)	.05	.47
Saw pediatrician at visit	178 (84.4)	128 (86.5)	50 (79.4)	.09	.19
Saw a female practitioner	149 (70.6)	99 (66.9)	50 (79.4)	.13	.07
Had ≥6 prior visits with practitioner	123 (58.3)	90 (60.8)	33 (52.4)	.08	.26
Substance use					
Alcohol use					
Any^c^	192 (91.4)	132 (89.2)	60 (96.8)	.12	.07
Median (IQR), d	3.0 (2.0-6.0)	3.0 (1.0-5.0)	4.0 (2.0-10.0)	−2.31	.02
Heavy episodic drinking^d^					
Any	70 (33.2)	46 (31.1)	24 (38.1)	.07	.32
Median (IQR), d	0 (0-2.0)	0 (0-1.0)	0 (0-3.0)	−0.93	.35
Cannabis use					
Any	106 (50.2)	73 (49.3)	33 (52.4)	.03	.68
Median (IQR), d	3.5 (2.0-15.0)	3.0 (1.0-15.0)	3.5 (2.0-13.8)	−0.67	.50
Used alcohol and cannabis	87 (41.4)	57 (38.5)	30 (48.4)	.09	.19
Any other drug use	8 (3.8)	6 (4.1)	2 (3.2)	.02	.76
CRAFFT score ≥2	59 (28.0)	41 (27.7)	18 (28.6)	.01	.90
Rode with driver who had been using alcohol or drugs in past 3 mo	56 (26.5)	35 (23.6)	21 (33.3)	.10	.15
Hung out with any friends who use alcohol or drugs	188 (89.1)	131 (88.5)	57 (90.5)	.03	.68
Substance-involved^e^					
Siblings^f^	82 (42.7)	59 (44.0)	23 (39.7)	.04	.57
Parents	35 (16.6)	25 (16.9)	10 (15.9)	.01	.86

Abbreviations: CRAFFT, car, relax, alone, forget, family or friends, and trouble; CSBI, computer-facilitated screening and brief intervention; IQR, interquartile range; NA, not applicable; UC, usual care.

^a^ Test statistics for continuous variables are from *t* tests (age) or Mann-Whitney *U* tests (days of use of alcohol, heavy episodic drinking, or cannabis); for categorical variables, Cramér *V* and associated *P* value, a measure of strength of association among categorical variables, are presented.

^b^ Owing to missing responses, n = 203; valid percentages are reported.

^c^ Owing to missing responses, n = 210; valid percentages are reported.

^d^ Heavy episodic drinking was defined using the National Institute on Alcohol Abuse and Alcoholism youth screening guide–recommended guidelines based on age and sex.

^e^ Percentage reporting any agree response to scale items from the Personal Experience Inventory assessing substance involvement of siblings or parents.

^f^ Owing to missing responses, n = 192; valid percentages are reported.

The prevention effect cohort consisted of 658 youths (CSBI, 478; UC, 180) who reported no use of alcohol or cannabis in the past 12 months at baseline. The CSBI group was younger and less likely to report hanging out with friends who used substances (eTable 1 in Supplement 2), and these variables were entered as control variables in all further analyses.

### Intervention Feasibility

In the intervention effect and prevention effect cohorts, youths in the CSBI group were more likely than youths in the UC group to report receiving advice about cannabis (ARRR, 1.36 [95% CI, 1.09-1.69]) and about not riding with an impaired driver (ARRR, 1.31 [95% CI, 1.09-1.57]) or driving while impaired (ARRR, 1.24 [95% CI, 1.03-1.50]) and to report receiving information about the health risks of alcohol use (ARRR, 1.22 [95% CI, 1.04-1.44]) and cannabis use (ARRR, 1.34 [95% CI, 1.09-1.65]) (Table 2; eTable 2 in Supplement 2). In the prevention effect cohort, there was significantly higher reported receipt of advice about avoiding alcohol use in the CSBI group compared with the UC group (ARRR, 1.30 [95% CI, 1.17-1.43]). In the intervention effect cohort, among 59 patients in the CSBI group with risk levels that indicated practitioners should try to bring them back for a follow-up visit, 27 (45.8%) reported being asked to return for a follow-up visit compared with 6 of 23 patients (26.1%) in the UC group, although this difference did not reach statistical significance. More than three-quarters of patients in the CSBI group in the intervention effect and prevention effect cohorts (42 of 55 [76.4%] and 141 of 178 [79.2%], respectively) reported receiving the Contract for Life. In the intervention effect cohort, the CSBI and UC groups did not differ significantly on receiving advice about alcohol use, ratings of the advice received, likelihood of following the advice, or satisfaction with the visit. In contrast, in the prevention effect cohort, patients in the CSBI group were significantly more likely than patients in the UC group to rate the information they received as excellent or good (ARRR, 1.13 [95% CI, 1.00-1.27]) and to be very likely to follow their practitioner’s advice (ARRR, 1.15 [95% CI, 1.00-1.31]) (eTable 2 in Supplement 2). However, they did not differ on level of overall satisfaction with the visit.

**Table 2.** Reports of Practitioner Counseling and Ratings of Their Visit Among Patients Who Reported Use of Alcohol or Other Drugs in the Past 12 Months at Baseline

Baseline Postvisit Assessment Measure	Total, No.	No./Total No. (%)		Adjusted Relative Risk Ratio (95% CI)^a^
		CSBI Group (n = 148)	UC Group (n = 63)	
Received advice				
Alcohol use^b^	211	105/148 (70.9)	36/63 (57.1)	1.21 (0.95-1.52)
Cannabis use^b^	211	122/148 (82.4)	37/63 (58.7)	1.36 (1.09-1.69)^c^
Not driving after using substance use^d^	201	119/141 (84.4)	41/60 (68.3)	1.24 (1.03-1.50)^c^
Not riding with impaired driver	211	129 (87.2)	43 (68.3)	1.31 (1.09-1.57)^c^
Received information on health and safety risks				
Alcohol use	211	132/148 (89.2)	47/63 (74.6)	1.22 (1.04-1.44)^c^
Cannabis use	211	117/148 (79.1)	40/63 (63.5)	1.34 (1.09-1.65)^c^
Excellent or very good rating of practitioner advice^e^	174	101/131 (77.1)	32/43 (74.4)	1.04 (0.85-1.26)
Very much likely to follow practitioner advice^e^	174	53/131 (40.5)	12/43 (27.9)	1.45 (0.85-2.46)
Very much satisfied with visit	211	90/148 (60.8)	36/63 (57.1)	1.04 (0.81-1.34)
Received Contract for Life^f^	88	42/55 (76.4)	5/33 (15.2)	5.04 (2.24-11.33)^c^
Asked to return for a follow-up visit^g^	82	27/59 (45.8)	6/23 (26.1)	1.93 (0.89-4.17)

Abbreviations: CSBI, computer-facilitated screening and brief intervention; UC, usual care.

^a^ Adjusted relative risk ratio with UC as the reference group. Logistic regression with generalized estimating equations adjusted for number of days of alcohol use in the past 12 months at baseline.

^b^ Advice to not start alcohol or cannabis use for youths who reported no prior use of alcohol or other drugs and advice to stop further use for youths who reported use of alcohol or other drugs in the past 12 months.

^c^ *P* < .01.

^d^ This question was asked of high school students only.

^e^ Among adolescents reporting receiving advice about alcohol or cannabis.

^f^ This question was added 9 months after recruitment started, so analysis includes only those who received this question.

^g^ This question was asked of patients considered to be at high risk only. Criteria for high risk were report of any use of alcohol or other drugs in the past 3 months in the screening and a yes response to any of the items on the CRAFFT (car [excluded in this instance], relax, alone, forget, family or friends, trouble) screening tool.

### Intervention Acceptability

In the total sample, youth feedback regarding their overall visit experience was highly positive, with 808 patients (92.9%) using words such as *excellent*, *fun*, *informative*, and *interesting*. Only 12 patients reported some discomfort, giving comments such as, “being asked about riding in cars with people that drink makes me nervous now,” or “thinking about someone driving drunk is upsetting.” Patients in the CSBI group tended to rate the CSBI psychoeducational content highly; median (IQR) scores were 5.0 (4.0-6.0) for useful, 5.0 (4.8-6.0) for convincing, 2.0 (2.0-4.3) for exaggerated, and 2.0 (1.0-2.0) for irritating. Fifty of 54 participating practitioners completed a follow-up questionnaire, and 44 (88%) rated the system as moderately or very useful, 40 (80%) reported that it increased their confidence in behavioral counseling, and 31 (62%) would recommend it to other practices, while 16 (32%) were undecided and 3 (6%) would not.

### Substance Use Outcomes

**Figure 2.**
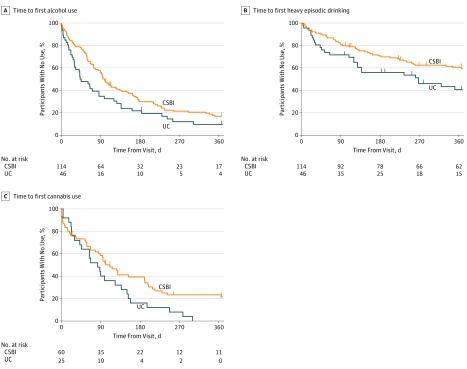
Kaplan-Meier Survival Curves for Time to First Use of Alcohol or Other Drugs During Follow-up Data presented are for youths who reported use of alcohol or other drugs in the past 12 months at baseline. Heavy episodic drinking was defined using the National Institute on Alcohol Abuse and Alcoholism youth screening guide–recommended criteria based on age and sex. Median (interquartile range) times to first use were 97 (51-222) days among the computer-facilitated screening and brief intervention (CSBI) group and 44 (21-143) days among the usual care (UC) group for any alcohol use (A); 366 (124-366) days among the CSBI group and 213 (51-366) days among the UC group for heavy episodic drinking (B); and 101 (33-226) days among the CSBI group and 83 (27-152) among the UC group for cannabis use (C). The crosses indicate censored observations within each study arm.

In the intervention effect cohort, adjusted hazard ratios from Cox proportional hazards modeling comparing time to first postvisit use between CSBI and UC groups, with adjustment for baseline group differences, were 0.69 (95% CI, 0.47-1.02) for alcohol use, 0.66 (95% CI, 0.40-1.10) for HED, and 0.62 (95% CI, 0.41-0.94) for cannabis use (adjusted hazard ratios <1 indicate that patients in the CSBI group tended to have longer time to first use compared with patients in the UC group) (Figure 2). In the prevention effect cohort, adjusted hazard ratios comparing time to first postvisit use between CSBI and UC groups, with adjustment for baseline group differences, were 0.87 (95% CI, 0.57-1.31) for alcohol use and 0.76 (95% CI, 0.44-1.32) for cannabis use (eFigure in Supplement 2). These results did not reach statistical significance, possibly because of low levels of substance use overall in this cohort.

### Riding Risk Outcomes

Among 99 patients in the total sample who reported riding risk at baseline, the CSBI group had an 18% lower risk than the UC group of reporting riding risk at the 6-month follow-up, but the difference was not statistically significant (ARRR, 0.82; 95% CI, 0.50-1.34). The effect size increased over time, with a 27% lower risk at 9 months (ARRR, 0.73; 95% CI, 0.44-1.19), although not statistically significant, and 42% lower risk at 12 months (ARRR, 0.58; 95% CI, 0.37-0.91) (Table 3). There were no meaningful differences between the CSBI and UC groups among the 769 participants who reported no riding risk at baseline.

**Table 3.** Self-reported Riding in the Past 3 Months With a Driver Who Had Been Drinking or Using Other Drugs Stratified by Reported Riding Risk in the Past 3 Months at Baseline^a^

Follow-up Time Point	No./Total No. (%)		Adjusted Relative Risk Ratio (95% CI)^b^
	CSBI Group	UC Group	
Riding risk at baseline^c^			
Baseline, No.	64	35	NA
6 mo	20/44 (45.5)	12/21 (57.1)	0.82 (0.50-1.34)
9 mo	16/39 (41.0)	13/21 (61.9)	0.73 (0.44-1.19)
12 mo	18/47 (38.3)	13/19 (68.4)	0.58 (0.37-0.91)
No riding risk at baseline^c^			
Baseline, No.	561	208	NA
6 mo	35/429 (8.2)	19/158 (12.0)	0.72 (0.43-1.23)
9 mo	33/419 (7.9)	10/163 (6.1)	1.33 (0.67-2.66)
12 mo	28/452 (6.2)	11/168 (6.5)	0.99 (0.50-1.99)

Abbreviations: CSBI, computer-facilitated screening and brief intervention; NA, not applicable; UC, usual care.

^a^ Because of technical issues with riding risk data collection at the 3-month follow-up, only data for 6-, 9-, and 12-month follow-ups are presented.

^b^ Logistic regression with generalized estimating equations adjusted for baseline group differences.

^c^ Analyses were conducted on the overall study sample of 869 patients; 1 participant had missing data for this measure.

## Discussion

Our results suggest that the CSBI system for youth substance use intervention and prevention is feasible and acceptable in primary care, and among patients who report substance use at baseline, effect size estimates were sufficient to warrant further testing. We also found sufficient effect size estimates for reducing youths’ risk of riding in cars with substance-impaired drivers, a behavior associated with a leading cause of youth mortality. Effect sizes among those who reported no substance use at baseline were smaller and not significant, perhaps owing in part to the low number of patients who began to use alcohol or other drugs during the 12-month follow-up.

We powered our parent study to assess the psychometric properties of the National Institute on Alcohol Abuse and Alcoholism–recommended approach to youth screening, not to demonstrate efficacy of the CSBI intervention. Nonetheless, we found a significant intervention effect on cannabis use, clinically relevant effect sizes for alcohol use and HED, and a significant decrease in riding with an impaired driver at the 12-month follow-up. However, sample sizes and effect sizes have been shown to be negatively correlated; therefore, given the moderate study sample used in these analyses, these effect sizes should be viewed cautiously.^30^ Additionally, we found that pediatric practitioners were able to deliver the brief intervention in only a few minutes, which bodes well for widespread adoption of the approach. Other studies of brief interventions in youth primary care have relied on standalone computer programs or trained therapists to deliver the intervention,^31,32^ which may be financially impractical for smaller pediatric group practices to implement.

The CSBI program was acceptable to the youth patients, who described it as excellent, fun, informative, useful, and convincing. Twelve patients reported some discomfort, largely in response to content about the dangers of riding with an impaired driver. The CSBI system’s feasibility has been supported by previous data published elsewhere.^19^ Of the 50 participating practitioners who completed a follow-up questionnaire, most of them rated the system as moderately or very useful, reported that it increased their confidence in behavioral counseling, and said they would recommend it to other practices. The greatest drawback reported was the time it took for the CSBI study protocol before the visit, which impeded clinic flow. Among suggestions for improvement was the need to integrate CSBI with the electronic health record.

### Limitations

This study had potential limitations. Using the same trained practitioners to deliver both the CSBI and UC intervention effect arms may have caused contamination of the UC arm, but we chose this design over practitioner randomization because our 2012 study^23^ found that practitioner characteristics are major covariates of the outcome, and we had only a modest total number of practitioners. We also considered randomization by practice, but this design requires too large a number of practices to be feasible under prevailing research funding limits. The only group difference we could ensure was the computer-based CRAFFT screen with immediate personalized feedback and 10 psychoeducational pages. We did find high rates of substance use–related counseling in our UC group compared with a national sample of US tenth graders, in which only 40% of adolescents who visited a physician in the past 12 months reported being advised about alcohol-related health risks and only 17% reported being advised to reduce or stop drinking.^7^ In our study, more than 60% of patients in the UC group reported receiving health risk information, and more than 57% of patients who reported prior substance use received advice to reduce or stop. Other factors that may have contributed to higher rates of counseling on use of alcohol or other drugs in our study include the Hawthorne effect^33^ caused by practitioners knowing they were being observed and all the participating sites being near Boston, Massachusetts, where our center has been active in substance use screening research, dissemination of results, and clinical teaching in the area for more than 15 years. Future studies should use more diverse, national samples. Additionally, we relied solely on reporting from patients of the intervention components received and did not obtain independent observations of practitioner behaviors. Despite these limitations, we still found estimates of effect size that would be clinically important and, for cannabis use and riding with impaired drivers, statistically significant.

Other study limitations include our reliance on self-reported data, although under confidential conditions, youths’ self-reports have been shown to be reliable and compare favorably with other forms of substance use detection.^34,35,36^ We were unable to examine effects on use of drugs other than cannabis owing to low prevalence. Additionally, we did not collect any data on parents’ or guardians’ views of the confidential CSBI system, including information on discussing the Contract for Life with their teenagers or viewing the Teen-Safe educational program. These data should be included in future research.

## Conclusions

The CSBI system showed sufficient feasibility and acceptability for implementation in busy pediatric practices to warrant further testing, and it showed promise for delaying postvisit alcohol and cannabis use, HED, and riding with impaired drivers among patients with prior risk. Future research should include larger samples, randomize by practitioner or practice, assess the contribution to the intervention’s effect by its various elements (eg, tablet computer informational pages, practitioner counseling), evaluate strategies to promote real-world dissemination, including integration of CSBI within electronic health record systems, and assess cost-effectiveness.

## Notes

Supplement 1. **Trial Protocol and Statistical Analysis Plan**

Supplement 2. **eTable 1.** Baseline Characteristics of Overall Group and by Study Arm Among Youths Reporting No Use of Alcohol or Other Drugs in the Past 12 Months at Baseline **eTable 2.** Youths’ Reports of Clinician Counseling and Ratings of Their Visit Among Those With No Reported Use of Alcohol or Other Drugs in the Past 12 Months at Baseline **eFigure.** Kaplan-Meier Survival Curves for Time to First Use During Follow-Up Among Participants Who Reported No Use of Alcohol or Other Drugs in the Past 12 Months at Baseline

Supplement 3. **Data Sharing Statement**
