# Supplementary material for: Effect of Computer-Based Substance Use Screening and Brief Behavioral Counseling vs Usual Care for Youths in Pediatric Primary Care: A Pilot Randomized Clinical Trial
Source: JAMA Netw Open. 2019 Jun 21;2(6):e196258. doi: 10.1001/jamanetworkopen.2019.6258 (PMC6593643; doi:10.1001/jamanetworkopen.2019.6258)
Supplement: Supplement 1. — Trial Protocol and Statistical Analysis Plan [file jamanetwopen-2-e196258-s001.pdf]

Title of Project: Computerized substance use Screening, Brief Intervention, and Referral to Treatment (C-SBIRT) for children and adolescents in primary care

This protocol reflects the combination of two separate protocols with the following titles:
1. Computerized Alcohol Screening for Children and Adolescents (cASCA) in Primary Care
2. Computer Adaptation of Screening, Brief MET Intervention to Reduce Teen Drinking

PI: John R. Knight, MD and Sion K Harris, PhD

Brief Summary/Abstract

The primary goal of this project is to develop a computerized screening program for primary care offices that is based on the NIAAA's new Alcohol Screening Guide for Children and Adolescents and assess its psychometric properties among nine- to 20-yr-old primary care patients. There is substantial evidence supporting the effectiveness of screening and brief intervention among adult primary care patients, primarily in the reduction of harmful drinking.[1-7] However, there have been few studies of alcohol screening and brief intervention conducted among adolescents seen in busy primary care settings.[8] This project will develop and validate a new computerized Alcohol Screening for Children and Adolescents (cASCA) program which incorporates the age-specific screening questions of the NIAAA Guide and includes the CRAFFT and AUDIT as secondary risk/problem assessments. We will add tobacco screening, because tobacco use is the leading cause of cancer-related mortality in the US, *as well as screening for marijuana and other drug use so as to create a comprehensive screening tool that includes all major substances that adolescents use*.[17]

Specific Aims/Objectives

The primary aims of this project relate to psychometric testing of the new computerized alcohol screening system based on the NIAAA guide. These include the following:

1. Assess the sensitivity, specificity, positive and negative predictive values of the 'any drinking/number of drinking days' and 'friends' questions of the cASCA in identifying past-year use as determined by the Timeline Follow-Back Calendar (TLFB), and for identifying any problem use, abuse or dependence as determined by the AUDIT, CRAFFT and a structured psychiatric diagnostic interview (computerized DISC-IV, Youth Version) at baseline (criterion validity).
2. Assess the test-retest reliability of the cASCA in measuring drinking frequency categories and in classifying patients into Low, Medium and High risk categories.
3. Assess the degree to which the 'any drinking/number of drinking days' and 'friends' questions predict drinking, "binge" drinking, and any problem use, abuse or dependence at 12-mos follow-up.
4. Assess the degree to which the 'any drinking/number of drinking days' item of the cASCA predicts drug use risk as measured by the TLFB (any use and frequency), tobacco use as measured by the Hooked on Nicotine Checklist (screen for potential nicotine dependence), drug use disorders as measured by the Drug Abuse Screening Test for Adolescents, and the degree to which it predicts other mental health problems as measured by the Pediatric Symptom Checklist-17 Youth self-report at baseline and 12-month follow-up.
5. Compare the psychometric properties of the cASCA across subgroups, including: age, gender, race/ethnicity, practice type, provider type, and patient risks (e.g., those with/without friends who drink); and explore the possible effects of the cASCA system on drinking during the 12-month follow-up period.

Additionally, the NIAAA guide recommends that providers deliver a brief intervention in response to the screening results. Therefore, a <u>*secondary aim*</u> *of this project will be to pilot-test a computer-facilitated Brief Intervention (BI) component using a randomized design comparing three groups: 1)*

screening with treatment as usual and all follow-up assessments [cASCA/TAU-ALL]; 2) screening with brief intervention and referral to treatment [Computerized Screening, Brief Intervention, Referral to Treatment (C-SBIRT) and; 3) a screening/TAU Minimal-Assessment arm in which participants will complete the screening and a minimal validation assessment at baseline, and no further assessments until the last follow-up at 12 months [cASCA/TAU-MA]. Because extensive and repeated assessment may itself help to change behavior, we have added this third arm to have a comparison group that minimizes this exposure. The BI component consists of patients viewing on the computer, immediately after the screening, their score and level of risk for a substance use problem, as well as several interactive pages of science and true-life stories about the health risks of substance use. Clinicians are then given the screen results and suggested talking points for a few minutes of brief counseling during the visit. Finally, patients identified as having problematic substance use, as defined by any positive response on the RAFFT items of the CRAFFT screen and any past-12-month substance use, will be given a "prescription" to complete a brief web-based computerized Motivational Enhancement Therapy (cMET) program and return for a follow-up visit in one month. The cMET program consists of exercises founded on Motivational Interviewing principles that are designed to enhance a teen's intrinsic motivation to change their substance use. Participants will be asked to complete this program in two sessions from home or another private computer sometime within the subsequent month. In this pilot study, we will assess feasibility and acceptability of the C-SBIRT system and estimate its effect size on underage drinking. Only participants ages 14-20 will be eligible to be randomized to receive the web-based cMET intervention, if screen-positive for problematic substance use. Participants ages 9-11 will only complete the screening program and be randomized to either all TAU with all follow-up assessments or TAU-MA. <u>Feasibility</u>: rates of clinician provision of brief advice and referral to the cMET program; teen completion of the cMET exercises, cMET sessions 1 and 2; <u>Acceptability</u>: adolescent and provider satisfaction; <u>Effect Size Estimate 1</u>: We will examine effects separately for participants who report any past-12-month alcohol use at baseline and those who do not, as we found in a prior study that the intervention effect can be quite different in these two groups. We will compare time to first alcohol use post-visit, and rates of alcohol use across the three study arms over the 12-month follow-up period. Effect Size Estimate 2: compare time to first use post-visit and rates of alcohol use across three study arms over a 9-month follow-up period for those screen-positive for problematic substance use: C-SBIRT + cMET vs. cMET only vs. Control - cASCA/TAU-ALL. <u>Hypothesis</u>: Effect Size Estimate 1: Among 12- to 18-yr-old primary care patients, those receiving C-SBIRT will have longer time to first alcohol use following their visit, lower rates of any alcohol use, days of alcohol use, drinks per drinking day, and days of heavy episodic drinking, than those receiving treatment as usual. Effect Size Estimate 2: Among 14-20-yr-old primary care patients, those receiving the cMET program will have longer time to first use post-visit, and lower rates of any alcohol use, days of alcohol use, drinks per drinking day and days of heavy episodic drinking, than those receiving treatment as usual.

Pilot-testing of cMET by Behavioral Health Providers*After initial pilot-testing which found low rates of cMET completion among participants asked to complete it on their own after leaving their baseline visit, we now propose to conduct a small formative research project to explore the feasibility/acceptability of use of the cMET program by behavioral health clinicians in the primary care setting to provide a brief intervention to patients at high risk for a substance use disorder. The cMET program could help to promote intervention implementation fidelity, and, with its computer interactivity, may enhance patient interest and engagement in the session. There will be two methods of recruitment: 1) The program will be introduced to eligible participants by their behavioral health provider, and completed during two clinical appointments with this provider, and 2) patients on the Research Participant Registry at the Adolescent Medicine Clinic who expressed willingness to be contacted for future studies and meet age-related eligibility criteria will receive information about the study and an invitation to participate via email. If interested and eligible, these patients will make an appointment with the behavioral health provider at the Adolescent Medicine Clinic and complete the program during two clinical appointments with this provider.We will examine the following as indicators of feasibility and acceptability: 1) the number of invited participants that consent to*

participate; 2) the rate of completion of each activity within each session; 3) the number that complete both program sessions; 4) participant feedback about the cMET program and their visit on post-session questionnaires; 5) time required to complete each session; and 6) a semi-structured debriefing interview with participating behavioral health providers at the end of the pilot project. We will use a continuous improvement process throughout the project using feedback from patients, providers, and our research staff to identify an implementation model that is feasible and acceptable, and shows promise for testing in a subsequent efficacy trial.

2. Background and Significance

The costs of treating substance use disorders in the US are estimated at over $6 billion annually[19], Substance use usually begins during adolescence; thus, by identifying and treating substance use problems early, substantial cost savings may be achieved and productive years of life gained. Universal screening of adolescents in primary care is a promising strategy for identifying substance use before more problems develop, and when brief interventions are more likely to be effective. There is substantial evidence supporting the effectiveness of screening and brief intervention among adult primary care patients, primarily in the reduction of harmful drinking [2-8]. It is still unknown, however, whether these findings can be generalized to younger patients, as there have been few studies conducted to date among adolescents in primary care[9]. Substance use is highly prevalent among U.S. adolescents and strongly linked to the leading causes of death: accidents, homicides and suicides.[1] It is also associated with a myriad of other serious health problems, making primary care medical offices promising venues for screening and early intervention.[1, 20-23] Greater than ¾ of adolescents see a physician yearly, and have trusting, longitudinal relationships with their providers.[24,25] Recognizing this opportunity, the American Academy of Pediatrics recommends that all adolescents receive substance use screening as part of routine care.[26, 27] However, adherence to this guideline is poor: less than 50% of pediatricians report screening all adolescents for substance use[28] and performed screenings are often of low quality, e.g., fail to use a structured screening or ask questions about impaired driving risks.[29,30, 31] Barriers identified by providers include lack of time and personnel to perform the screening, unfamiliarity with screening tools, lack of training in how to manage positive screens, and lack of effective interventions.[15] The problem is compounded by the current paucity of scientific evidence. While recommending alcohol Screening, Brief Intervention and Referral to Treatment (SBIRT) for adults, the U.S. Preventive Services Taskforce found that the "evidence is insufficient" to recommend for or against SBIRT for adolescents.[9] Changing this situation requires the development and testing of new medical office strategies that increase the frequency and quality of screenings, assist providers in managing screening results, do not impede clinic flow or add to providers' time, and are shown to be effective in reducing patients' substance use.[32, 33] A validated computerized Alcohol Screening for Adolescents and Children (cASCA) based on the NIAAA alcohol screening and brief intervention guide for youth will provide an additional tool to address this need and has the potential to reduce youth alcohol and drug use and associated health-risk behaviors, prevent serious consequences, and greatly lessen the later burdens of high treatment costs and human suffering**.**

3. Preliminary Studies

The PI and his investigative team have conducted a portfolio of research studies on medical office screening for adolescent substance abuse over the past 15 years. [10-16] We developed the CRAFFT[22] by combining similar items from the extant research literature, added the word "ever" to each question to enhance sensitivity to detect use, and expanded the scope of screening to include both alcohol and drugs, and tested the items among 99 adolescent clinic patients ages14-18. We arranged the six items based on the first letters of key words in each item (Car, Relax, Alone, Forget, Family/Friends, Trouble), created the mnemonic acronym CRAFFT to aid recall of the items, and simplified the scoring (score equals the number of "yes" responses). Convergence between the CRAFFT and scores on a similar but longer measure, PICS (Personal Involvement with Chemical Scale[23]), was high (r = 0.84). We then assessed criterion validity in a general adolescent clinic sample of 538 14-18 yr olds using two criterion standards: the 17-item Substance use/Abuse scale of the Problem-Oriented

Screening Instrument for Teenagers [24], a measure designed to assess problem SU and the potential need for treatment, and the Adolescent Diagnostic Interview (ADI),[25] a structured diagnostic interview that yields DSM-IV diagnoses of alcohol/drug abuse and dependence. Sensitivity/specificity analyses indicated that a CRAFFT score of 2 was the optimal cutpoint for identifying any problem use (sensitivity=0.76; specificity=0.94; positive predictive value [PPV]=0.83; negative predictive value [NPV]=0.91), any disorder (sensitivity=0.80; specificity=0.86; PPV=0.53; NPV=0.96) and dependence (sensitivity=0.92; specificity=0.80; PPV=0.25; NPV=0.99). The CRAFFT score also demonstrated severity scale-like properties with the PPV increasing linearly with higher CRAFFT score. Validity was not significantly affected by age, gender, or race/ethnicity.

We next measured the prevalence of positive screens among >2000 12-18-yr-old patients from a practice-based research network in New England,[26] assessed teens' preferences for substance use screening (they favored computer or paper questionnaires),[27] identified common barriers to provider screening, designed a computer-facilitated Screening and Brief Advice (cSBA) protocol in response, and compared it to Treatment As Usual (TAU) among 2096 adolescents in the USA and 539 in the Czech Republic.[28] The cSBA protocol doubled provider screening and brief advice rates in the USA and quadrupled them in the CZR. The cSBA system also showed promise for reducing adolescent substance use, particularly alcohol use in the USA and marijuana use in the CZR. As a result of this work, the CRAFFT screening and advice algorithm has become the standard of care for pediatric primary care practice and has been widely integrated into both print and online clinical encounter forms. It has been published in professional pediatric policy statements[29]and practice guides[30-32], in the Bright Futures toolkit and encounter forms[31], in textbooks[33, 34], and review articles[5, 35]. It has been translated into more than 10 other languages including Chinese, Japanese, Haitian Creole, Czech[3] and Spanish.

However, in one research study we found that some adolescents were confused by the questions if they had never used substances. We therefore added lifetime and past-12-month use questions and instituted a skip pattern for all but the CAR question in CRAFFT. We are currently conducting a small study to validate a brief-screen frequency item for drug use. Since the publication of the NIAAA Guide, we have planned to modify our approach to make the two screening algorithms completely consistent with one another. In this project, we will validate both the peer use and drinking frequency quick-screen items for alcohol use, which is more harmful to youth than all other drugs combined and for which our computerized SBA system showed large and significant effects.

4. Design and Methods

a. Study Design

This is a prospective validation study which assesses test-retest reliability and concurrent and predictive validity over a 12-month follow-up period. In addition, this is a pilot randomized controlled trial of a computer-facilitated brief intervention (C-SBIRT) to reduce alcohol use rates among adolescent primary care patients. All participants will complete the screening and baseline validation measures for the psychometric study. Participants ages 12-20 will then be randomized into three study arms for the randomized controlled trial: 1) screening with treatment as usual [cASCA/TAU] (n=595); 2) screening with the computer-facilitated Brief Intervention, Referral to Treatment [C-SBIRT] (n=850); and 3) a screening/TAU Minimal-Assessment arm (n=255) in which participants will complete the screening and a minimal validation assessment at baseline, and no further assessments until the last follow-up at 12 months [cASCA/TAU-MA] (see study flow diagram). Because extensive and repeated assessment may itself help to change behavior, we have added this third arm to have a comparison group that minimizes this exposure. Participants ages 9-11 will be randomized into cASCA/TAU and cASCA/TAU-MA only. All participants will complete the final 12-month follow-up while only those in the first two groups will complete the 3, 6, and 9 month follow-up assessments. In addition, to assess test-retest reliability, we will consecutively invite 150 participants to complete a re-test of the screening program through secure web-based access from home or other private computer

within 2 weeks of baseline. Test-retest participants will be asked to report on the same period at both baseline and re-test, so as to avoid their covering two different time periods in the test and retest. We will exclude participants assigned to the "minimal-assessment" (cASCA/TAU-MA) and brief intervention arms from being invited to do the re-test, the first to minimize the number of assessments that the minimal-assessment arm completes, and the second because receipt of an intervention could change participants' responses due to potential social desirability bias. This study will involve participants completing the consent/assent process and screening before the visit, which we anticipate will take no more than 10 min total, and the remaining baseline assessment after the visit. We have found in prior studies that this does not interfere with clinic flow. We also have a strong track record of working with clinic sites to meet their unique needs and our study recruitment goals. Participants will receive a $15 merchandise gift card for completion of the baseline visit, $10 for the retest, $10 each for the 3-, 6-, and 9-month assessments, and $15 for the 12-month follow-up, or a maximum total ranging from $30 (baseline and 12-month only) to $70 (all possible assessments, including re-test).

From September 15, 2016 – January 27, 2017, all participants were invited to complete the Baseline Questionnaire, for which they were compensated $15. However, only High-Risk participants (determined by screening to have problematic substance use) will be followed up and invited to complete 3-, 6- and 9-month questionnaires. Participants will receive $10 for the 3- and 6-month questionnaires and $15 for the 9-month questionnaire. We no longer administered the retest.Thus, participants recruited during this phase can receive up to $50 for completing all portions of the study.

High Risk participants were randomized into the cASCA/TAU group, into the C-SBIRT/cMET group or into the cMET-only group. Compensation for participants who complete the cMET program is as follows: $15 gift card if they complete Session 1 in the clinic during the Baseline visit, or $10 if they complete Session 1 as home; $10 for Session 1; $15 for the one-month follow-up visit with their clinician, for a total of $35-40 in addition to the$50 for the other study activities.

Initial Pre-Test Phase: *Before beginning the full study, we will first pre-test our computerized screening and brief intervention program, as well as all study assessments in a small group of youth ages 9-18 in order to assess clarity, comprehension, and functionality, and we will refine all materials as needed. We will invite up to 15 youth, or 5 from each age group: elementary school (ages 9-11), middle school (ages 12-14), and high school (ages 15-18). We will recruit them from local schools and from participating clinics.*

Randomization Scheme: In this multi-site study, the computerized Study Management System will randomly assign participants within each site to one of three study arms using a weighted block scheme developed and successfully implemented in our previous studies.[25] Block factors of gender, age group, and past-3-months heavy episodic drinking will be implemented, with randomly permuted block sizes, and weighted to the 35% cASCA/TAU-MA, 15% cASCA/TAU-ALL, and 50% C-SBIRT, arm-proportion objectives of the study.[69] This system ensures balance across participant demographic factors and the three conditions in order to maximize efficiency in group comparisons, while maintaining investigation blindness prior to assignment.

From September 15, 2016 – January 27, 2017, participant follow-up was conditioned on their risk level for a substance use disorder, as determined by screening. All participants were invited to complete the Baseline questionnaires. However, only High Risk participants (those determined to have problematic substance use) will be invited to complete 3-, 6- and 9- month follow-up questionnaires. The study groups will be designed as follows:

1) cASCA/TAU-MA – Treatment-as-Usual with Baseline Questionnaire only.

2) cASCA/TAU-ALL – Treatment-as-Usual with Baseline Questionnaire, 3-, 6- and 9-month follow-ups (to be completed on the computer or over the phone).
3) C-SBIRT + cMET – Computerized Screening and Brief Intervention, with Baseline Questionnaire, 3-, 6- and 9-month follow-ups (to be completed on the computer or over the phone). These patients will view their risk level and the brief intervention, as described above. In addition, they will be invited to complete the cMET program during the next month (described in more detail below). These participants will be asked to return for a follow-up visit with their clinician about one-month after the baseline visit.
4) cMET only – Baseline Questionnaire, 3-, 6- and 9-month follow-ups (to be completed on the computer or over the phone). In addition, these partcipants will be invited to complete the cMET program during the next month (described in more detail below). These participants will be asked to return for a follow-up visit with their clinician about one-month after the baseline visit.

b. Patient Selection
We will recruit approximately 30 primary care providers from offices affiliated with the New England Partnership for Substance Abuse Research (NEPSAR), and a youth sample, using age by gender stratification, of 1700 nine- to 20-y/o primary care patients coming for routine primary care at one of the participating physicians' offices. The broad age range covers the screening groups in the NIAAA guide – late elementary school (ages 9-11), middle school (ages 12-14), and high school (ages 15-20). We are extending our recruitment age group to include those 19- to 20 years old as they constitute a pediatric primary care patient population with a high prevalence of hazardous alcohol use, and would thus benefit from a brief MET intervention addressing problematic alcohol use. Eligible patients must have an email address and internet access at home, school, or library, and must provide informed assent/consent. We will exclude any who are unable to read or understand English, living away at college at the time of the recruitment visit, unavailable for computer/telephone follow-ups, or judged by the provider to be medically or emotionally unstable at time of visit. Adolescents do not have to have ever used substances to participate. NEPSAR is a practice-based research network founded by Dr. Knight in 2003. NEPSAR has recently affiliated with the Pediatric Practices of Children's Hospital Boston (PPOC), comprising 75 pediatrician offices in eastern Massachusetts with more than 200 physicians who care for more than 300,000 infants, children, and adolescents. We have also added the Children's Hospital Primary Care Center, which includes 30 providers and 42,000 visits per year, and with an estimated 5000 patients 9-18 years old. The greatest threat to validity of studies such as this one is slow recruitment and inadequate sample size. Over the course of our previous research we have successfully recruited more than 30 outpatient practices, 100 primary care providers, and more than 6,000 adolescents. Practices have included the spectrum of places adolescents receive routine care: adolescent clinics, general pediatrician offices, family medicine offices, and school-based health centers. We will work with providers who see on average at least eight nine- to 18-y/o patients/week and provide informed consent to participate in this study. We will exclude medical students, residents, and fellows from the study as we have found in a prior study that the provider brief advice intervention was less effective when delivered by less experienced clinicians.[17]

We will advertise the study through posters and brochures in clinic waiting rooms as a "Substance Use Prevention Study", clearly indicating youth need never to have used alcohol or drugs to be eligible. *The site Research Assistant (RA) will compare the clinic appointment list with an age-by-gender recruitment table, flag the records of potential participants and mail introductory letters about one month before their visit describing the study purpose and procedures and confidentiality protections to both adolescents and parents/guardians.* The RAs will then call them within two weeks prior to the visit, briefly describe the study and invite interested youth to arrive at the clinic at least 30 minutes before their scheduled visit. Upon arrival, we will obtain informed assent/consent. *If time allows, we will also approach eligible patients in the waiting room before their visit.* For those <12 years-of-age we will obtain parental consent and adolescent assent. For those 12-17 years-of-age we

will obtain informed assent but are requesting a waiver of parental permission. We will obtain informed consent from all 18-20 year old participants.

c. Study Treatments and Exposures/Predictors

Upon assent/consent, all participants will self-complete the computerized Alcohol Screening for Children and Adolescents (cASCA) prior to seeing their provider (see attached Study Flow Diagram). The cASCA program begins with age-dependent screen questions which determine participant risk level. *Participants randomized to the C-SBIRT arm will then view a) their screening score and risk level, and (b) several interactive web pages of science and true-life stories about substance use risks, all of which we have found in previous studies to take less than 5 minutes. Additionally, (c) providers will be given their screening results and bullet points to guide 2-3 minutes of brief counseling, and, (d) if the patient has problematic substance use, as defined by any positive response on the RAFFT items of the CRAFFT screen and any past-12-month substance use, s/he will receive a referral to a 2-session computer motivational enhancement therapy (cMET) intervention available on the secure website* www.myvyou.com. *Patients can complete these sessions in the clinic or on a computer at home. A stylized picture or "avatar" of the provider will guide the patient through the activities on the program. Patients randomized to the cASCA/TAU or cASCA/TAU-MA conditions will complete only the screening questions before their visit, and their provider will not receive the computerized screen results. However, many providers already conduct screening and provide brief advice and arrange follow-up as part of routine practice so participants in the TAU conditions may receive some of the same information during the visit as those in the C-SBIRT group.*

Description of the cMET Intervention: *Motivational Enhancement Therapy (MET) is a type of structured intervention infused with the principles and techniques of Motivational Interviewing (MI), a client-centered, semi-directive counseling style that is characterized by collaboration, compassion and accurate empathy, acceptance and affirmation, and evocation of a client's own values and ideas ("MI Spirit"). The flow of a MI-based intervention typically consists of first "engaging" a client in a working alliance based on trust and partnership, assessing and reviewing the concern, followed by a "focusing" of the alliance towards client-centered change goals ("setting the agenda"), using an "evoking" communication style throughout (e.g., asking open-ended questions, using reflective listening, "rolling with resistance" and avoiding argument). Throughout the consultation, periodic summarization of the discussion helps to reinforce the client-centered nature of the discussion, and information/advice can be offered when the client invites it or gives permission. This relational foundation then serves as the platform on which the following core MI/MET strategies (or change mechanisms) are implemented: 1) helping clients review their values and life goals, and pros and cons of continuing substance use, with the aim of "developing a discrepancy" between their goals and their substance use, and of creating a "decisional balance" tilted toward recognition of the costs of use; 2) eliciting clients' own "change talk" or reasons, and self-efficacy, for behavior change; and 3) creating a specific change plan for each goal. These MI strategies align with widely-known theoretical models of behavior influence, i.e., the Health Belief Model, Social Cognitive Theory, and Trans-Theoretical Model (TTM) of Change, in which key mediators of behavior change include perceptions about problem severity and the value of harm reduction ("importance"), perceptions about self-efficacy for change ("confidence") and barriers to action, and expectancies about the outcomes of the change. These factors contribute to the "stage of change," as outlined in the TTM, at which a client may present (e.g., "pre-contemplation" or "action"), which can both moderate the effectiveness of MET, and change in response to MET.*

Our MET approach involves sequential completion of highly-structured therapeutic tools over two sessions over 2-4 weeks. To develop our initial clinician-delivered MET we made substantial adaptations to strategies proven effective among adults, including assessment and feedback, negotiation and goal setting, decisional balance, creating a goals-behavior discrepancy, and follow-up and reinforcement. Our adaptation involved creating structured tools and exercises that cover risks and problems more relevant to adolescents (e.g., school issues, parent and family concerns), are fun

and engaging, and that provide concrete prompts and visualization of options as teens may have more difficulty with abstract thinking. For example, the "Values Card Sort" is one such tool which was designed to help develop a discrepancy between teens' goals/values and their substance use behavior. In this exercise, adolescents are given a stack of cards on which are written different "goals" or "values" such as "To go to college" or "To have a lot of money." They then sort the cards into "extremely important to me," "not important to me," and "not sure" piles. The clinician then reviews the "extremely important" values with patients in a discussion about whether substance use aligns or conflicts with their values.We adapted the clinician-delivered MET to be self-administered on the computer (cMET). We have given the cMET program the "brand" name "myVYou," a more teen-friendly name that denotes its use as a tool for "re-view-ing" one's goals and behaviors.

Below is an outline of the exercises included in the myVYou program, and descriptions of some activities and language used:

Session 1

Introductory Pages: myVYou begins with a welcome page providing an introduction to the program and navigation instructions. This is followed by a section of pages called "What Do I Like?" where teens can personalize myvYOU by choosing an avatar to represent themselves and identifying interests, hobbies, pets, sports, extracurricular activities and future college/career plans. The last few introductory pages establish an understanding of treatment by 1) explaining the purpose of myVYou ("to explore together the impact that tobacco, alcohol, or drugs may be having on your life and health"); 2) explaining the limits of confidentiality ("all information is confidential unless we detect a serious safety risk"); and 3) explain the ground rules of the therapeutic alliance ("any decision to change is up to you"). The clinician's avatar appears on each screen and gives instructions and feedback as the patient goes through the program.

Exercise #1: "What's Important to Me?" Patients are presented with a set of swirling "cards," each with a brief phrase describing a value or goal (i.e. "To get into a good college," "To be trustworthy," "To do well in sports") and one-by-one "grabs" them with their mouse and places them in one of three possible piles – "Very important to me," "Somewhat important to me," "Not important to me." They then indicate whether their substance use helps or interferes with reaching each of the goals they chose as "Very important."

Exercise 2: "Pros & Cons" A teen is shown balancing on a skateboard while, one at a time, paper airplanes fly in from either side and open up to reveal a "Pro" of use (i.e. "Drinking helps me relax," "Marijuana helps me forget my problems") or a "Con" (i.e. "I get sick or feel hung over when I drink," "Marijuana makes me lazy."). (Fig. 3) Patients check those that apply to them and the airplanes land on the ground in the "Pro" pile or the "Con" pile. The clinician avatar comments on the relative size of the piles ("Wow, it looks like the cons outweigh the pros.") Pros and cons are presented separately for each substance.

Exercise 3: "Draw the Line" Patients see a female teen with a can of spray paint standing in front of a wall with a line painted on it. The clinician avatar asks them to choose a number between 0-10 indicating how important it is for them to change their use, after which the girl paints the number on the wall. The clinician avatar then asks "Why didn't you choose a lower number?" and patients choose a suggested response or type in their own. The next screen has a male teen asking how confident they are that they can change their use, again prompting a choice between 0-10.

Exercise 4: "What Have I Experienced?" Patients are presented with scenes of a city street, a school, inside a house, and a park and field. Clicking on a red square by a specific picture opens a box with a brief phrase describing a scenario, i.e. "Fighting with parents," "Getting kicked off a sports team or

club." Patients click a box to say they have experienced this, and at the end of the exercise they are shown all the scenarios they endorsed.

<u>Exercise 5:</u> "Take 2" In this tool, the clinician avatar invites patients to make a "Change Plan." Patients are then shown a set of colored fan blades which open up to reveal lists of activities and people that could support their plan. They check a box for those they would find helpful or type in their own idea. Those not ready to make a change plan are asked to consider possible situations that would indicate a need to change.

<u>Session 2</u>

"Take 2" Review: Patients review their initial change plan, enter information about successes and setbacks, have an opportunity to enter revised goals and plans and again choose people and activities to support their plans. As in Session 1, those not ready to make a change are asked to consider possible situations that would indicate a need to do so. Clinicians are again notified by myVYou of their patient's session completion, they review the completed work, and send a response back to the patient."

Participants who are randomized to the cMET intervention will receive a $15 gift card for completing Session 1 in the clinic, or a $10 gift card for completing Session 1 outside of the clinic. They will receive $10 for completing Session 2 outside of the clinic and $15 for returning to the clinic for a 1-month follow-up appointment with their clinician. cMET intervention participants can therefore receive an additional $35-40 in gift cards for their participation.

Pilot-testing of cMET by Behavioral Health Providers

This formative research project will involve patients in the Boston Children's Hospital Adolescent/Young Adult Medical Practice that are referred for behavioral health services or recruited through the Research Participant Registry at the Adolescent Medicine Clinic. Participant eligibility requirements include: 1) being ages 12-20 yrs, 2) able to read and understand English, 3) used any substance in the past month, and 4) medically and emotionally stable at time of visit as determined by their clinician. During a visit, participating behavioral health providers will ask whether an eligible patient would be interested in learning about a research study in which they will try out and give feedback about a computer program (called "myVYou") over 2 visits, and then will provide them with an information flyer. If the patient is interested, the provider will ask him/her to arrive 15 minutes early to their next appointment so that a research study assistant can meet them, provide more information about the study and confidentiality protections, and obtain written assent from patients ages 12-17 or informed consent from patients aged 18-20. We currently have an IRB-approved waiver of parental consent for patients aged 12-17 years.

During the initial visit for all participants, providers will conduct substance use screening using a tablet computer program, entering responses directly into the program. This information will automatically be imported into the myVYou program which will be tailored to address each patient's substance(s) used. At each of the two study visits, the provider will log-in to the secure study website (www.myVYou.org) and guide the patient through the completion of each exercise, with discussion and reflection at each step. Participants will be scheduled for the second session to occur within 4 weeks. Prior to the second visit, providers will be able to log-in to the myVYou website to review patients' responses to exercises during the first session. At completion of each visit, participants will complete a brief paper questionnaire about their impressions of the program and visit, and receive a $30 merchandise gift (Amazon or Target) card (for a total of $60 for both visits).

d. Definition of Primary and Secondary Outcomes/Endpoints

Our primary outcomes/endpoints will be the following: 1) cASCA screening sensitivity, specificity, positive and negative predictive values in identifying current (baseline), and risk for future (12 months later), tobacco, alcohol, drug use prevalence, frequency and severity (as measured by the Timeline Follow-Back Calendar interview, the Hooked on Nicotine Checklist, the Alcohol Use Disorders Identification Test, the CRAFFT screen, the NIMH Diagnostic Interview Schedule for Children-IV, and the Drug Abuse Screening Test for Adolescents); and 2) test-retest reliability measured within 2 weeks.

In addition, our secondary outcomes will be 1) determination of C-SBIRT's acceptability and feasibility for use with adolescent primary care patients and providers, and 2) estimation of an initial effect size comparing C-SBIRT vs. TAU rates of past-90-day alcohol use at 3-, 6-, and 9-month follow-ups. Specifically, we will examine rates of any initiation or cessation of alcohol use, days of alcohol use, drinks per drinking day, and days of heavy episodic drinking. Secondary outcomes will include initiation and cessation of any alcohol use, "binge" drinking, and substance-related driving (for those of driving age) and riding (all ages) at each follow-up.

Pilot-testing of cMET by Behavioral Health Providers
We will examine the following as indicators of feasibility and acceptability: 1) the number of invited participants that consent to participate; 2) the rate of completion of each activity within each session; 3) the number that complete both program sessions; 4) participant feedback about the cMET program and their visit on post-session questionnaires; 5) time required to complete each session; and 6) a semi-structured debriefing interview with participating behavioral health providers at the end of the pilot project. We will use a continuous improvement process throughout the project using feedback from patients, providers, and our research staff to identify an implementation model that is feasible and acceptable, and shows promise for testing in a subsequent efficacy trial.

e. Data Collection Methods, Assessments and Schedule

Recruitment Data: Using a computer-based recruitment form, we will record the date of the visit and patient's date of birth (the computer calculates age and then deletes the DOB); gender; grade in school; race/ethnicity; socioeconomic status (number of parents living in the household and highest educational level of parent(s)); the name of the visit provider; the reason for the visit (i.e., well care, urgent care, follow-up visit, other), and patient status (i.e., established vs. new patient for that practice). The form will note any reasons for study exclusion, the response to the invitation to participate, and any reasons for refusal, and ask those who are ineligible and those who refuse for permission to keep these data. We will later use these data to identify possible differences between the study population and groups of excluded and refusing patients.

Contact Information: The contact information, including at least three telephone numbers (home, work, cell, parent cell/work, or friend cell/home), mailing address, and email addresses. The study management system will store the contact information in a separate encrypted "logbook" file, which will not be part of the study data file, but will be used to schedule and conduct follow-up measurements.

Baseline Assessment Battery: Following completion of the cASCA, all C-SBIRT and cASCA/TAU-ALL participants will complete a baseline study assessment battery consisting of the following widely-used, validated measures described below. We have selected economical measures and scales to promote the likelihood of study participation, minimize the risk of the study interfering with clinic flow, and to make follow-up data collection easier, thereby aiding study retention. All measures will be self-administered on the computer, with an audio-assisted version available (with headphones), except for the Timeline Follow-Back Calendar interview which will be conducted by trained research assistants. Participants are free to skip questions they do not want to answer. They will be prompted twice by the computer to complete a skipped item, and then

allowed to proceed to the next item if they try to skip a third time. The cASCA and baseline assessment battery will be administered in a private location at the clinic.

- *Brief Substance Use History questionnaire (3-6 min completion time depending on extent of use):*
  - The assessment battery will begin with a brief self-administered, branching questionnaire that separately records any (yes/no) lifetime, past-12-months, and past-3-months use of alcohol, tobacco, marijuana, and other drugs. In addition, for each substance ever used, we will ask for age of first use.
  - We will ask the Marijuana Source Items in order to examine changes in marijuana source which may impact accessibility and usage rates. We will also ask questions about electronic vapor products because there is more and more reported use of these products among participants.
- *Timeline Follow Back Calendar (TLFB) (5-15 min completion time depending on use):* Trained research assistants (RA) will then confidentially conduct with all participants a past-12-months Timeline Follow-Back (TLFB) interview assessing the number of days used, and amount used on those days, of alcohol, tobacco, marijuana, non-medical use of Rx/OTC medications, and other drugs. We considered separately recording each illicit drug (e.g., MDMA, cocaine), but rejected this because of low use rates in our previous studies and the additional time required. The TLFB is a reliable and valid calendar-based data collection method, generally conducted by personal interview, used extensively in substance use studies of both adults and adolescents to measure self-reported frequency and quantity of substance use during a defined time period.[16, 60-64] By utilizing a calendar and specific memory aids to enhance recall, it can generate relatively precise estimates of use of each substance assessed. We will use the TLFB as the criterion standard to test the validity of responses to the screening item on drinking days, and will be able to determine the number of "binge" drinking days (as defined by the NIAAA age- and gender-specific guidelines). One or two sips of alcoholic drinks taken during family or organized religious events (e.g., communion) will not be recorded. RA training to conduct the interview will include reading of instructional materials, observation of a videotaped demonstration, and a series of practice interviews with observation and feedback until a prescribed level of competence is achieved. Throughout the recruitment period, study investigators will periodically observe and use structured forms to rate RAs on interview technique. RAs who fail to achieve an acceptable rating will receive retraining and increased monitoring.
- *Substance Use Severity measures (15-20 min depending on severity):* Patients reporting any *alcohol* use on the TLFB will also complete the 1) Alcohol Use Disorders Identification Test[13, 65-70] (10 items) and 2) CRAFFT screen[10, 12, 13] (6 items) to identify any problem use (with or without a diagnosis); and the 3) Alcohol Abuse/Dependence Module of the computerized, audio-assisted, self-interview NIMH Diagnostic Interview Schedule for Children-IV (Voice C-DISC IV), Youth Version[71, 72] to identify alcohol abuse or dependence. These three measures will serve as criterion standards for examining the sensitivity, specificity, positive predictive value, and negative predictive value of the 2-item cASCA in predicting problem alcohol use or alcohol disorders. We chose the C-DISC-IV because of its appropriateness for youth as young as 9 yrs old. Additionally, participants reporting any *tobacco* use on the TLFB will complete the 10-item Hooked on Nicotine Checklist[73-76], while those reporting any *drug* use will complete the 28-item Drug Abuse Screening Test-Adolescent (DAST-A)[77, 78], both brief measures of substance use severity.
- *The Importance/Confidence of Change questions and the Contemplation Ladder (1-2 minutes):* These measures will allow us to assess whether our cMET intervention affects patient ratings of the importance of changing their substance use behavior, their level of confidence to change their behavior, and their readiness to change.
- *Personal Consequences of Substance Use questions (1-2 minutes):* Previous studies of brief interventions to address adolescent substance use found that substance use-related consequences tended to decline more than substance use consumption outcomes. This measure will assess substance use-related consequences.
- *Perceived Availability/ Perceived Risk of Harm (3-5 minutes):* These questions come from the national Monitoring the Future survey and will allow us to compare our sample's data to national

data.

- *Riding/Driving Risk (5 min):* All participants will complete a 10-item measure on past-90-day Riding with an impaired driver, or Driving while impaired (if driving age). If any Riding risk is reported, participants will be asked about the characteristics of the impaired driver (Someone who lives with you? (yes/no), A member of your family? (yes/no), An older adult? (yes/no), Someone close to your age? (yes/no)), but purposely avoid asking any identifying information to protect the privacy of parents/legal guardians or other potential secondary subjects.
- *Peer Chemical Environment (2 min):* To assess construct validity of the "friends' drinking" item on the screening questions, we will administer a 5-item self-report measure derived from the widely used Personal Experience Inventory (PEI)[45]. Respondents are asked to agree or disagree (4-point Likert response scale) with 5 statements about their peers' alcohol and drug attitudes and involvement (e.g., "The kids I hang around with think it's okay for kids to drink alcohol" and "Some kids I hang around with have trouble at school due to using drugs or alcohol").
- *Sibling Substance Use (2 min)**:*** Also from the PEI, these 4 items assess the alcohol and drug involvement of brothers and sisters (e.g., "I have a brother or sister who uses alcohol or drugs with me").
- *Family Pathology (5 min)**:*** This 8-item scale from the PEI measures family problems and parents'/guardians' alcohol/drug use (e.g., "I have a parent who gets drunk or high" and "I have a parent whose use of alcohol or other drugs worries me").
- *Behavioral and Mental Health:* To assess the sensitivity/specificity of the screen in identifying youth with behavior problems and other psychiatric disorders (e.g., externalizing disorders, internalizing disorders, ADHD), we will administer the Pediatric Symptom Checklist – 17, Youth self-report version. This is a 3-minute 17-item measure that is a validated and widely-used screening measure for child and adolescent psychiatric disorders.
- *Modified Heartland Forgiveness Scale and questions to identify spirituality/religiosity (2-4 minutes):* In a prior cross-sectional study, of about 300 adolescent primary care patients, we found that, among multiple religiosity and spirituality measures, forgiveness had the strongest association with decreased adolescent alcohol use. The current study, with its much larger sample size, will allow us to determine replicability of this finding, as well as examine the effect prospectively.

Post-Visit Checklist: To assess the degree to which screening and brief intervention is being implemented by providers during a visit, participants in all groups will be asked to complete a brief 2-min post-visit checklist immediately following their provider encounter documenting what questions about alcohol and other drugs they were asked by their provider, and what advice or counseling they received.

Test-Retest: To assess test-retest reliability of the cASCA items, during the first three months of recruitment we will recruit up to 150 participants *from the cASCA/TAU-ALL arm only* (block-stratified by age group [9-11, 12-15, 16-18, 19-20] and gender) to complete the cASCA (reporting on the same 12-month time period as at baseline) by logging into the secure study website no later than two weeks from their baseline assessment. Research assistants (RA) will contact participants by email and phone one week after their baseline visit prompting them to complete the retest within the week. The email will provide the secure link to the questionnaire, and the unique user ID and password for logging in. If a participant does not have an email address, s/he will be called with the log-in information. We will make reminder calls to participants who have not yet completed the retest within 3 days of the email. We will continue sampling and recruitment until we have 150 retest completions. Participants will also complete a computerized self-administered past-12-months TLFB at this time which will allow us to assess agreement between RA interview and computer self-administration modes. If we find high agreement between responses on the computerized self-administered TLFB

and the baseline RA interview, we will use the computerized self-administered TLFB rather than a phone interview for follow-up data collection. The self-administered mode offers greater privacy, and prior studies have indicated that adolescents may under-report substance use in phone interviews compared to computer-assisted self-interviewing (CASI) or self-administered paper-pencil surveys.[80-83] If we find inadequate agreement between the RA interview and computer self-administered modes, we will use RA interviews to collect follow-up TLFB data to maintain mode homogeneity.

Beginning September 15, 2016, we will no longer be administering the Test-Retest to participants.

Follow-up Assessments: We will collect data on alcohol, tobacco, and other drug use frequency/quantity during the 12-month follow-up period using the TLFB. The TLFB administration mode (computer self-administered or phone interview) used will be determined by the results of the initial comparison of the interview and computer self-administered modes (see Test-Retest section above). In addition to the TLFB, all of our follow-up assessments to reflect our baseline assessment battery, so that data can be compared across all measurement time points. If we use phone interviews, we will use procedures to minimize risks to confidentiality and/or validity in telephone interviews (e.g., setting advance appointments so adolescents can be in a private place), and RAs will begin the phone interview with questions that assess level of privacy (e.g., “Where are you right now? Who else is there with you? Can anyone overhear our conversation?”) to complete a past-90-day TLFB, as well as the Riding/Driving Risk items, at 3, 6, and 9 months. If a participant misses one of these follow-ups, the next assessment will ask about the entire time period from the last TLFB completion; e.g., if a participant does not complete the 3-month TLFB, the 6-month TLFB will ask about the entire 6-month time period since the baseline assessment. The 3-, 6-, and 9-month follow-up assessments will help us to maintain ongoing contact with the participants so as to enhance their retention in the study out to 12 months, and will also help to maximize accuracy and completeness of data over the 12-month follow-up by giving us multiple opportunities to reach a participant during the follow-up period. The every-3-months assessments will also allow us to collect data, whenever possible, using shorter recall periods. We have previously shown 90 days to be a reliable recall period for adolescents (Levy S et al., ACER, 2004;28(8):1256-1261). At 12 months follow-up, participants will complete the same assessment battery as at baseline. The contact schedule for 3, 6, 9 and 1 year follow-ups is outlined below:

Day 0: Initial Patient email and text message about follow-up assessment due

If no response, the following are sent until assessment is completed:

Day 3: Reminder Patient email and SMS; Parent email

Day 5: RA phone call

Day 9: Reminder Patient email and SMS

Day: 11: RA phone call

Day 15: Reminder Patient email and SMS; Parent email

Day 17: RA phone call

Day 21: Last reminder Patient email and SMS

Day 23-30: RA phone calls as needed

Beginning September 15, 2016, we will shift to a 3-, 6- and 9-month follow-up schedule for High-Risk patients; Low- and Medium-Risk patients will not be followed up. Efforts to retain these participants will adhere to the same follow-up contact schedule as outlined above.

Pilot-testing of cMET by Behavioral Health Providers
Participants will be be asked to complete the cMET program during two clinic visits with a behavioral health provider. They will receive a reminder phone call prior to each scheduled visit. If required visits have not been scheduled or a scheduled appointment is missed, attempts to follow-up with the participants will be made via alternating emails or phone calls every 4 days for a total of three weeks, at which point they will be considered lost to follow-up. Once both cMET sessions and the follow-up questionnaires are complete, no additional follow-up will be required.
User experience and acceptability/feasibility:
Adolescents. *Participants receiving cMET will complete a brief user feedback survey about the program immediately after completion of each cMET session, which assesses their user experience and satisfaction with the program (e.g., clarity of instructions, ease of use, overall liking of the design/animation, respectfulness, usefulness, most positive and negative aspects, suggestions for improvement). In addition, we will record any questions or problems users have as they go through the cMET program, and the program will automatically record 1) time to complete each exercise and the session as a whole; 2) which MET tools/components were completed; and 3) all the selections made by the participant during the exercise including their importance and confidence ratings; their change goals, if any; and the number and types of strategies and supports for change included on the change plan. At the 9-month measurement we will also ask adolescents to give overall feedback on helpfulness, and satisfaction with, the cMET system as a whole, including the provider-patient communication system and monthly messages.*

Providers. *At the end of recruitment, we will ask providers to complete a brief exit questionnaire which assesses their demographics (gender, type of provider [attending, nurse practitioner, physician assistant, etc.], years of practice), experience with using the cASCA and cMET programs, how they tended to use the screening results, if at all; the advice they usually gave their patients, if any; and their usual follow-up plan for patients in the TAU arm.*

Pilot-testing of cMET by Behavioral Health Providers
As noted above, adolescent participants will complete a substance use screening questionnaire during their visit with the behavioral health provider. They will also complete demographics questions, and a brief user feedback paper questionnaire after completion of each study visit, which assesses their user experience and satisfaction with the program (e.g., clarity of instructions, ease of use, overall liking of the design/animation, respectfulness, usefulness, most positive and negative aspects, suggestions for improvement). The program will automatically record 1) time to complete each exercise and the session as a whole; 2) which MET tools/components were completed; and 3) all the selections made by the participant during the exercise including their importance and confidence ratings; their change goals, if any; and the number and types of strategies and supports for change included on the change plan. We will also conduct a semi-structured debriefing interview with participating behavioral health providers after each cMET session and at the end of the pilot project.

g. Study Timeline

<u>*Phase 1:*</u> *During Phase 1 of this project (10/1/13-5/31/14), we will develop all necessary study materials, including all computer programs, such as the c-ASCA program and study management system. In addition, we will identify and work with all of our partner clinic sites to create a feasible implementation plan, obtain all IRB approvals needed for the study, and hire and train all research staff. Finally, we will conduct a pre-testing of all study materials with a small sample of 15 youth (5 elementary, middle, and high school students) prior to beginning fully study implementation.*

Phase 2: Before recruitment begins, we will hold orientation meetings at each practice, explain the purpose and procedures of the study, and obtain informed consent. We will provide a one-session training to providers at each practice that includes a demonstration of the computer program, a review of a sample Provider Report, and viewing a video that shows a pediatrician giving 2-3 min of brief advice to a patient using the provided talking points. Patient recruitment and measurement will then continue throughout Phase 2 (6/1/14-1/31/17).

5. Adverse Event Criteria and Reporting Procedures

Given the nature of the study, we anticipate few if any adverse events. We could receive complaints related to the time spent on study measurements or visits, or undesired clinical outcomes (alcohol/drug use or other risk behaviors), which would not be directly due to the interventions under study. If we determine during the course of the study that an adolescent or someone else is at serious risk of harm, we will notify the provider per the Safety Protocol. (See below.) We do not anticipate any serious adverse events, such as medical complications or deaths, as a result of this study. It is possible that a death or occasional emergency room visit or hospitalization will occur, however, it is highly unlikely that any of these will be study-related. Nonetheless, we will establish an ad-hoc study Data and Safety Monitoring Board (DSMB), to review any serious alcohol-related motor vehicle crashes, injuries, emergency room visits, hospitalizations, arrests, or deaths, should they occur. Dr. Grace Chang, an addiction psychiatrist with expertise in substance abuse treatment, will chair the DSMB, and the DSMB will include representatives from the participating NEPSAR practices. We will report any serious adverse events immediately to Dr. Chang for review and to the Children's Hospital Boston Committee on Clinical Investigations (IRB) and the IRB of the involved site. We will summarize AEs and SAEs in the annual report to NIH, but notify NIH project officers immediately if the DSMB or IRB judges any SAE to be study-related.

Safety Protocol: We have established a structured safety protocol to further protect participants' welfare. We define an **emergency** safety risk as one where the participant reports an intention to harm self or others. In an emergent situation, the RA will immediately end the study procedures and notify a medical care provider that the participant may be a danger. We anticipate few emergency safety risks in the proposed study; however a patient could spontaneously reveal suicide risk which would trigger the safety protocol. We define a **non-emergency** safety risk as one in which the participant reports high levels of substance use, such as drinking twice the binge amount for their age category, daily use of alcohol or marijuana, use of a drug other than marijuana on more than six occasions, or three or more episodes of Driving/Riding, during the past-3-months. In non-emergency situations, the RA may allow the participant to complete the measurement but must notify the provider, site PI, or Dr. Knight within 1 working day. The provider or PI will review the case and determine if further evaluation or treatment is necessary. We will not ask questions about child abuse or neglect during our study measurements or provider brief advice sessions. However, if a participant spontaneously reports this information to study personnel, we will report the information to the provider, who will conduct a further assessment, and notify child protection authorities as appropriate and required by state law. We will review this safety protocol with all site PIs, providers and site personnel at the study orientation and during monthly Steering Committee conference calls.

We will train all RAs in the safety protocol during initial and periodic training-review meetings. As part of the informed assent/consent process, the RA will inform youth that, in the event of a safety risk, we will notify the provider or site Principal Investigator (PI), who will speak with them further. The provider or site PI may in turn decide to inform the participant's parent(s) of safety risks or the need for specialty treatment. We inform youth that we cannot make absolute assurances or provide them with specific guidelines on parental notification. We explain that these decisions must be individualized and based on the best clinical judgment of their provider at the time. We do assure youth that we will make every effort to inform them whenever parental notification of a safety risk is required, and to include them in a discussion regarding the exact details to be released. Although parents could legally demand the release of study information for minor participants, in our previous research with over

5000 adolescents, we have had only one such request. In this instance the PI, Dr. Knight, met with the parent, explained the risks of a breach of confidentiality, and offered the parent a comprehensive substance use evaluation as an alternative, which the parent accepted. We have obtained a Certificate of Confidentiality from the National Institutes of Health to provide additional protection for participants. This certificate protects the investigators against being compelled to release confidential information to any law enforcement personnel.

Pilot-testing of cMET by Behavioral Health Providers
The same safety procedures described above will be followed. If an RA becomes aware of any potential safety concerns, s/he will report it within 1 working day to the patient's behavioral health provider, the behavioral health provider's supervisor, and study PI's (Knight and Harris). If any safety concerns arise during the clinical visit with the behavioral health provider, the provider will notify her supervisor and the study PI's. The providers and Dr. Knight will review the case and determine if further evaluation or treatment is necessary. If a participant spontaneously reports information about child abuse/neglect to a study staff person, we will report the information to the provider who will conduct a further assessment, and notify child protection authorities as appropriate and required by state law. We will review this safety protocol with providers at a study orientation for the Adolescent/Young Adult Medical Practice.

6. Data Management Methods
We will use a Study Management System (SMS) which uses tablet computers for data collection at each site with periodic data upload to a server located within the secure firewall of Boston Children's Hospital (BCH). The system has the following features: (a) A menu driven user interface specific to the investigation that provides an overview of the study and each study participant's status; (b) Protocol management and reminder prompts to promote protocol adherence (e.g., prompts for RAs to make reminder calls and posttest appointments); (c) Data integrity features (range and logic checks) and context sensitive help; (d) Status variables that permit ongoing tracking of participant status in the study; (e) Edit reports for missing, out-of-range and illogical responses; (f) Regular reports of subject accrual and pre-specified aggregate data; (g) Use of transactional databases until data for particular forms or participants are finalized and ready for transfer to the study master file; (h) Secure master files; (i) Daily server back-up, data security and confidentiality with access to both the client and server and permitted functions (e.g., making changes to data) controlled by administrator password; and (j) Automatic audit trail capability that tracks changes to data and keeps track of system users. The Data Manager (DM) encrypts all tablet computers with HIPAA compliant, BCH-managed PGP Universal Server software. The system automatically uploads all study related data except for identifying information weekly to the BCH server using encrypted SSL protocol VPN tunnels. The server generates reports for recruitment, retention and IRB progress reports. The server also manages study manager software updates for the client tablet computers. We will monitor recruitment and retention monthly with this system, which rapidly detects any practice sites that are outliers, and work intensely with any sites that are falling behind study targets.

7. Quality control methods
We will conduct a study orientation with providers prior to beginning recruitment in which we will present the NIAAA screen guide, demonstrate cASCA and review the study protocol. *Patients* will complete an adherence checklist (Post-Visit Checklist) at the end of the medical visit that records whether each part of the screening and brief advice was performed, and its perceived quality. We will periodically analyze results from the Post-Visit Checklists to identify study sites and providers who do not adhere to the protocol; offer retraining and monitoring; and drop from the study any who do not improve.

The CeASAR Director of Data and Technology will monitor participant recruitment and produce regular recruitment reports which will be reviewed at monthly study meetings. We will hold initial

training meetings with site providers and Research Assistants before recruitment starts, and hold follow-up meetings to ensure adequate recruitment.

8. Data Analysis Plan:
We will initially calculate univariate statistics (frequencies, means, medians) to characterize the demographics, substance use, and environmental risk profiles of our study sample. To evaluate potential selection and non-response bias, we will compare the demographics of participants and those who were eligible but did not participate in the study, when data are available, and compare the demographics of participants who were retained through the 12-months follow-up and those who were lost to follow-up.

Psychometric analyses
To address our psychometric study aims, we will conduct the following analyses:
Aims 1, 3, and 4: For elementary school-aged youth (9-11 yrs), we will examine validity of the lifetime "any drinking" item by comparing participant responses on the cASCA to responses on the Brief Substance Use History (BSUH) questionnaire (lifetime use and age of initiation of use, if any) in the confidential study assessment battery. We will calculate sensitivity, specificity, positive predictive values (PPV) and negative predictive values (NPV) for the cASCA item in identifying any lifetime use. For youth ages12-20 yrs, we will examine validity of the cASCA past-year "number of drinking days" item in two ways. We will compute intra-class correlation coefficients (ICC)[86] assessing agreement between the two continuous variables of total past-year number of days reported on cASCA and on the TLFB. In addition, we will recode past-12-months TLFB data into a dichotomous 0 days/1 or more days of past-year-drinking variable and calculate sensitivity and specificity of the cASCA item to detect any past-12-month drinking on the TLFB. We will also create 4 additional dichotomous TLFB variables based on the NIAAA cumulative number-of-days risk thresholds or "cut-points"[87], i.e., $\leq$5 vs. $\geq$6 days, $\leq$11 vs. $\geq$12 days, $\leq$23 vs. $\geq$24 days, and $\leq$51 vs. $\geq$52+ days. We will compute sensitivity/specificity of the cASCA item to predict these thresholds of use on the TLFB for the sample overall, and for each NIAAA-specified age group (9-11, 12-15, 16, 17, 18-20 yrs). Finally, we will assess the degree to which the cASCA "any drinking/number of drinking days" item predicts any *problem alcohol use* as defined by a screen-positive on either the AUDIT (score $\geq$2[13]) or CRAFFT (score $\geq$2[12]) or meeting criteria for an alcohol use disorder on the C-DISC-IV (PPV for problem use); an *alcohol use disorder* as determined by the C-DISC-IV (PPV for *disorder*); any past-year *tobacco use* and *tobacco problem* as identified by the TLFB and the HONC (score $\geq$1 indicates potential nicotine dependence); any past-year *drug use* and *drug problem* as identified by the TLFB and DAST-A; and any other *behavioral/psychiatric problem or disorder* as identified by the Youth DPS. We will compute both *concurrent* risk estimates (baseline data) and *future* risk estimates (12-months follow-up data) for each. For the cASCA "friends' drinking" item, we will recode responses to the 5-item Peer Chemical Environment scale into a single dichotomous "any agree" (respondents agreed to any of the 5 statements) vs."all disagree" (respondents disagreed with all statements) variable. We will examine *convergent* validity by computing a Cohen's kappa coefficient[88, 89] for the level of agreement between the cASCA "friends" item and the concurrent dichotomous Peer Chemical Environment variable at baseline. For 14-20 yr olds, we will recode responses to the cASCA "friends' usual number of drinks" item into two categories, "has binge-drinking friends" (based on the NIAAA-specified binge drinking thresholds[90]) and "no binge drinking friends." Using the same binge drinking thresholds, we will also compute an "any past-year binge drinking"/"no past-year binge drinking" variable using TLFB responses. We will then compute sensitivity, specificity, PPV, and NPV of the cASCA friends' binge-drinking item to predict participants' past-year binge drinking at baseline, and at 12-months follow-up.

Aim 2: We will compute Cohen's kappas for evaluating 1-2 week test-retest agreement on cASCA responses regarding any lifetime drinking (ages 9-11 yrs), any past-year drinking (12-20 yrs), any drinking friends (9-20 yrs), and any binge drinking friends (14-20 yrs). We will calculate ICC's for agreement on past-year number of days of drinking and number of usual drinks by friends, as well as agreement in risk categorization (low, medium, high) as defined by the NIAAA guide.

Aim 5: We will examine the degree to which the psychometric properties of the cASCA items are consistent across adolescent demographic subgroups (gender, age group, race/ethnicity) and practices. We will compute our reliability and validity statistics described above for each subgroup and study site, and compute 95% confidence intervals around each statistic, using bootstrap resampling to generate them when necessary.[91, 92] Non-overlapping 95% CI's will indicate significant differences in the performance of the cASCA items between groups.

Intervention acceptability/feasibility/efficacy
We will conduct all group analyses using intention-to-treat groups. The analysis of C-SBIRT intervention effects will include data for 12- to 18-year-old patients only, as we did not test the intervention among 9-11 year-olds, and we are recruiting 19- and 20-year-olds solely for the cMET pilot study. We will compare our experimental groups on all baseline measures to assess whether the study randomization scheme produced equivalent groups. Any variables meeting a p-value<0.20 in baseline group comparisons will be entered as covariates in multivariable modeling of the intervention effect. We will then use backward stepwise elimination of non-significant variables in all our multivariable modeling (using an inclusion criteria of p≤0.10) to produce the most parsimonious final models. We will adjust our variance estimates to account for our multi-site cluster sampling design using Generalized Estimating Equations (GEE), and observed differences will be considered statistically significant at p<0.05. We will compare results of our outcomes analyses conducted with and without imputed missing data. We will use predictive multivariable regression modeling to obtain imputed data.

Feasibility/Acceptability: *We will assess feasibility and acceptability of the C-SBIRT system, and particularly with the cMET program, by measuring patient and provider engagement and satisfaction. The cMET system tracks patient and provider login, session duration on a page-by-page basis, and logs user responses and messages sent and received to/from patients and providers. This will enable us to determine the percentage of patients assigned to cMET that actually utilize the system, and to what degree they complete each page. For all myVYou patients, we will use the clinic schedule to track how many appointments they have with their clinician or a social worker over the course of the study's 1-year period. This will provide us with additional information to determine feasibility and acceptability of the program. We will analyze patient and provider satisfaction results both quantitatively and qualitatively to identify features, content that are particularly helpful or in need of improvement, and to identify common themes regarding use of the system.*

Pilot-testing of cMET by Behavioral Health Providers
We will calculate the participation rate among those eligible and invited, characterize participant demographics, and examine response frequencies for items on the user feedback paper questionnaire completed after each study visit. We will also 1) calculate the mean (±SD) time required to complete each exercise and each session as a whole; 2) examine the rate of completion of each session; and 3) characterize the selections made by participants during the exercise including their importance and confidence ratings; their change goals, if any; and the number and types of strategies and supports for change that were selected.

Estimate of effect size**: We will examine the intervention effect on alcohol use (primary outcome) and use of cannabis and other drugs (secondary outcome), we will conduct the following analyses stratified by whether they reported past-12-month use of the substance at baseline: 1) using the Timeline Follow-back data for the full 12-month follow-up period, we will conduct Cox Proportional Hazard modeling to compute adjusted hazard ratios for comparison of* ***time to first use *of alcohol (or drugs) post-visit between those receiving C-SBIRT vs. TAU (those receiving all follow-up assessments). In this multivariable analysis, we will control for any variables on which the groups differed at baseline. Because of the availability of calendar-based use data across the full 12 months of follow-up, we have the unique ability to conduct this time-to-event analysis. 2) we will also use*

multiple logistic regression modeling with GEE (to account for within-site clustering) to compute adjusted relative risk ratios comparing group rates at follow-up of any drinking, any heavy episodic drinking, any tobacco, cannabis, or other drug use, and any substance-related driving (for those of driving age) and riding (all ages) risk with any identified potential confounders entered as covariates in the model. We will also examine the intervention effect on frequency and intensity of alcohol use at follow-up, as indicated by the past-90-days number of drinking days, and the average number of drinks per drinking day. As these variables are likely to have data distributions that are highly skewed and overdispersed, we will conduct GEE modeling specifying a negative binomial distribution and log link to compute adjusted incidence rate ratios.

9. Statistical Power and Sample Considerations

Aims 1, 3, 4 and 5: We will obtain measures of sensitivity, specificity, PPV and NPV of the first two questions of the cASCA. Based on prior studies, [12-14] we estimate that sensitivity and specificity of the frequency item for identifying past year use will be very high (≥ .90). For identifying problem use (including an alcohol disorder) we estimate specificity to be much lower (> .50)., With these assumptions, 75% retention at 12 months and 95% confidence intervals, a low and high estimate of the minimum detectable difference (MDD) can be made.[93] We note that a proportion of .50 yields a theoretical maximum (or worst case) MDD compared to proportions closer to 0.0 or 1.0. These MDD values (see table 1 below) are adequate to identify important age and gender differences in the sensitivity, specificity, PPV and NPV of these two questions.

Table 1: Minimum Detectable Differences in Sensitivity, Specificity, PPV and NPV for various group sizes and values.

Minimum Detectable Difference (MDD) for:	Group Size	MDD for Low Value (.50 is "worst case")	MDD for High Value (.90)
Baseline by gender	500	4.4%	2.6%
Baseline by year of age	100	10.0%	6.0%
12-Month by gender	375	4.9%	3.1%
12-Month by year of age	75	11.3%	7.3%

Aim 2: We will assess the test-retest reliability of the first two cASCA questions using the kappa and ICC. Based on data from a prior reliability study using similar questions,[16] we estimate kappa agreement for use/no use to be ≥ .80 with precision (e.g. ± 95% CI) ≥ .07. For the reliability of the frequency categories of the two questions, based on similar questions in the prior study, we estimate the ICC to be ≥ .90 with precision ≥ .03. These values, if realized, will provide strong evidence for the reliability of these questions.

10. Study Organization:
John R Knight MD, Principal Investigator; Sion Kim Harris PhD CPH, Co-Principal Investigator; Lon Sherritt MPH, Director of Research Technology; Melissa Weiksnar MBA, Project Manager; Sarah Bliss BA, Erin Gibson MPH, Project Coordinators; Sarah Copelas BA, Clinical Research Coordinator; Jessica Tauber BA, Kateryna Kuzubova MA, Jesse Boggis BA, Madeline Beauregard BA, David Butterworth BA, Jill Finlayson BA, Jordan Levinson, Research Assistants; Paula Carroll, Intern.

Pilot-testing of cMET by Behavioral Health Providers
<u>Current team</u>: John R Knight MD, Principal Investigator; Sion Kim Harris PhD CPH, Co-Principal Investigator; Lon Sherritt MPH, Director of Research Technology; Erin Gibson MPH, Project Coordinator; Jordan Levinson, Research Assistant.

References

1. Eaton DK, Kann L, Kinchen SA, et al. Youth Risk Behavior Surveillance -- United States, 2005. *MMWR.* 2006;55(SS-5):(SS-5).
2. Fleming MF, Barry KL, Manwell LB, Johnson K, London R. Brief physician advice for problem alcohol drinkers. A randomized controlled trial in community-based primary care practices. *JAMA.* Apr 2 1997;277(13):1039-1045.
3. Bertholet N, Daeppen JB, Wietlisbach V, Fleming M, Burnand B. Reduction of alcohol consumption by brief alcohol intervention in primary care: systematic review and meta-analysis. *Archives of Internal Medicine.* 2005;165(9):986-995.
4. Grossberg PM, Brown DD, Fleming MF. Brief physician advice for high-risk drinking among young adults. *Ann Fam Med.* Sep-Oct 2004;2(5):474-480.
5. Fleming MF, Mundt MP, French MT, Manwell LB, Stauffacher EA, Barry KL. Brief physician advice for problem drinkers: long-term efficacy and benefit-cost analysis. *Alcohol Clin Exp Res.* Jan 2002;26(1):36-43.
6. World Health Organization Brief Intervention Study Group. A cross-national trial of brief interventions with heavy drinkers. *American Journal of Public Health.* 1996;86:948-955.
7. Fleming MF, Balousek SL, Grossberg PM, et al. Brief physician advice for heavy drinking college students: a randomized controlled trial in college health clinics. *Journal of Studies on Alcohol and Drugs.* 2010;71:23-31.
8. Babor TF, McRee BG, Kassebaum PA, Grimaldi PL, Ahmed K, Bray J. Screening, Brief Intervention, and Referral to Treatment (SBIRT): toward a public health approach to the management of substance abuse. *Subst Abus.* 2007;28(3):7-30.
9. U.S. Preventive Services Taskforce. Screening and Behavioral Counseling Interventions in Primary Care to Reduce Alcohol Misuse. Available on-line: http://www.ahrq.gov/clinic/uspstf/uspsdrin.htm. Accessed 10/22/2009. In: Agency for Healthcare Research and Quality US Department of Health and Human Services, ed2004.
10. Knight JR, Shrier LA, Bravender TD, Farrell M, Vander Bilt J, Shaffer HJ. A new brief screen for adolescent substance abuse. *Archives of pediatrics & adolescent medicine.* Jun 1999;153(6):591-596.
11. Knight JR, Goodman E, Pulerwitz T, DuRant RH. Reliabilities of short substance abuse screening tests among adolescent medical patients. *Pediatrics.* Apr 2000;105(4 Pt 2):948-953.
12. Knight JR, Sherritt L, Shrier LA, Harris SK, Chang G. Validity of the CRAFFT substance abuse screening test among adolescent clinic patients. *Arch Pediatr Adolesc Med.* Jun 2002;156(6):607-614.
13. Knight JR, Sherritt L, Harris SK, Gates EC, Chang G. Validity of brief alcohol screening tests among adolescents: a comparison of the AUDIT, POSIT, CAGE, and CRAFFT. *Alcohol Clin Exp Res.* Jan 2003;27(1):67-73.
14. Knight JR, Sherritt L, Gates E, Harris SK. Should the CRAFFT substance abuse screening test be shortened? *J Clin Outcomes Manag.* 2004;11(1):19-25.
15. Van Hook S, Harris SK, Brooks TL, et al. The “Six T’s”: Barriers to screening teens for substance abuse in primary care. Paper presented at: Pediatric Academic Societies 2005; Washington, DC.
16. Levy S, Sherritt L, Harris SK, et al. Test-retest reliability of adolescents' self-report of substance use. *Alcohol Clin Exp Res.* 2004;28(8):1236-1241.
17. Harris S, Csemy L, Sherritt L, et al. Computer-facilitated substance use screening and brief advice for teens in primary care: an international trial. *Pediatrics.* Jun 2012; 129(6): 1072-1082.
18. Substance Abuse and Mental Health Services Administration Office of Applied Studies. *The DASIS Report: Alcohol and Drug Services Study (ADSS) Cost Study.* Rockville, MD2004.
19. U.S. Department of Health and Human Services. *The Health Consequences of Smoking: A Report of the Surgeon General.* Atlanta, GA: U.S. Department of Health and Human Services,

Centers for Disease Control and Prevention, National Center for Chronic Disease Prevention and Health Promotion, Office on Smoking and Health;2004.
20. Adrian M, Barry SJ. Physical and mental health problems associated with the use of alcohol and drugs. *Subst Use Misuse.* Sep-Nov 2003;38(11-13):1575-1614.
21. Chang G, Sherritt L, Knight JR. Adolescent cigarette smoking and mental health symptoms. *J Adolesc Health.* Jun 2005;36(6):517-522.
22. DuRant RH, Smith JA, Kreiter SR, Krowchuk DP. The relationship between early age of onset of initial substance use and engaging in multiple health risk behaviors among young adolescents. *Arch Pediatr Adolesc Med.* 1999;153(3):286-291.
23. Kulig JW. Tobacco, alcohol, and other drugs: The role of the pediatrician in prevention, identification, and management of substance abuse. *Pediatrics.* 2005;115(3):816-821.
24. Newacheck P, Brindis CD, Cart C, Marchi K, Krwin C. Adolescent Health Insurance Coverage: Recent Changes and Access to Care. *Pediatrics.* August 2, 1999 1999;104(2):195-202.
25. Harris SK, Woods ER, Sherritt L, et al. A youth-provider connectedness measure for use in clinical intervention studies. . *Journal of Adolescent Health.* 2009;44(2 Suppl):S35-36.
26. Elster A, Kuznets N, eds. *AMA Guidelines for Adolescent Preventive Services (GAPS)*. Baltimore: Williams & Wilkins; 1994.
27. Hagan J, Shaw J, Duncan P, eds. *Bright Futures Guidelines for Health Supervision of Infants, Children, and Adolescents: Third Edition*. Elk Grove Village, IL: American Academy of Pediatrics; 2008.
28. American Academy of Pediatrics. *Periodic Survey of Fellows #31: Practices and Attitudes Toward Adolescent Drug Screening.* Elk Grove Village, IL: American Academy of Pediatrics, Division of Child Health Research;1997.
29. Millstein SG, Marcell AV. Screening and counseling for adolescent alcohol use among primary care physicians in the United States. *Pediatrics.* 2003;111:114-122.
30. Hingson RW, Heeren T, Winter MR. Age at drinking onset and alcohol dependence: age at onset, duration, and severity. *Arch Pediatr Adolesc Med.* 2006;160(7):739-746.
31. Sung M, Erkanli A, Angold A, Costello EJ. Effects of age at first substance use and psychiatric comorbidity on the development of substance use disorders. *Drug & Alcohol Dependence.* 2004;75(3):287-299.
32. Butler SF, Chiauzzi E, Bromberg JI, Budman SH, Buono DP. Computer-Assisted Screening and Intervention for Alcohol Problems in Primary Care. *Journal of Technology in Human Services.* 2003;21(3):1-19.
33. Daniels V, Somers M, Orford J, Kirby B. How can risk drinking amongst medical patients be modified? The effects of computer screening and advice and a self-help manual. *Behavioural Psychotherapy.* 1992;20(1):47-60.
34. Belamarich PF, Gandica R, Stein RE, Racine AD. Drowning in a sea of advice: pediatricians and American Academy of Pediatrics policy statements. *Pediatrics.* Oct 2006;118(4):e964-978.
35. Thomas JW, Grazier KL, Ward K. Economic profiling of primary care physicians: consistency among risk-adjusted measures. *Health Serv Res.* Aug 2004;39(4 Pt 1):985-1003.
36. Bock B, Niaura R, Fontes A, Bock F. Acceptability of computer assessments among ethnically diverse, low-income smokers. *American Journal of Health Promotion.* 1999;13(5):299-304.
37. Deehan A, Templeton L, Taylor C, Drummond C, Strang J. Are practice nurses an unexplored resource in the identification and management of alcohol misuse? Results from a study of practice nurses in England and Wales in 1995. *Journal of Advanced Nursing.* 1998;28(3):592-597.
38. Israel Y, Hollander O, Sanchez-Craig M, et al. Screening for problem drinking and counseling by the primary care physician-nurse team. *Alcoholism: Clinical & Experimental Research.* 1996;20(8):1443-1450.
39. Knight JR, Harris SK, Sherritt L, et al. Adolescents' preferences for substance abuse screening in primary care practice. *Substance Abuse.* 2007;28(4):107-117.

40. Harris SK. Computer-facilitated substance use screening and brief advice for teens in primary care: an international trial. In Press. 2011; http://ceasar.org/isbirt/materials/cSBA_Outcomes_Manuscript.pdf.
41. Schwartz RH, Wirtz PW. Potential substance abuse. Detection among adolescent patients. Using the Drug and Alcohol Problem (DAP) Quick Screen, a 30-item questionnaire. *Clinical Pediatrics.* 1990;29(1):38-43.
42. Rahdert ER, ed *The Adolescent Assessment/Referral System Manual.* DHHS Publication No. (ADM)91-1735 ed: US Dept Health Human Services (PHS) Alcohol, Drug Abuse, and Mental Health Administration; 1991.
43. Babor T, de la Fuente J, Saunders J, Grant M. *AUDIT: The Alcohol Use Disorders Identification Test: Guidelines for use in primary health care.* Geneva, Switzerland: World Health Organization;1992.
44. Winters K, Stinchfield R, Fulkerson J, Henly G. Measuring alcohol and cannabis use disorders in an adolescent clinical sample. *Psychol Addict Behav.* 1993;7(3):185-196.
45. Winters K, Stinchfield R, Henly G. *The Personal Experience Inventory test and manual.* Los Angeles: Western Psychological Services;1988.
46. Winters K, Stinchfield R, Henly G. Further validation of new scales measuring adolescent alcohol and other drug use. *J Stud. Alcohol.* 1993;54:534-541.
47. Winters K, Henly G. *Adolescent Diagnostic Interview (ADI) Manual.* Los Angeles: Western Psychological Services;1993.
48. Knight JR, Harris SK, Sherritt L, et al. Prevalence of positive substance abuse screens among adolescent primary care patients. *Arch Pediatr Adolesc Med.* 2007;161(11):1035-1041.
49. American Academy of Pediatrics CoSA. Screening, brief intervention, and referral to treatment for pediatricians. *Pediatrics.* 2011;128(5):e1330 -e1340.
50. Jellinek M, Patel BP, Froehle MC, eds. *Bright Futures in Practice: Mental Health -- Volume I. Practice Guide*. Arlington, VA: National Center for Education in Maternal and Child Health; 2002.
51. Jellinek M, Patel BP, Froehle MC, eds. *Bright Futures in Practice: Mental Health -- Volume II. Tool Kit*. Arlington, VA: National Center for Education in Maternal and Child Health; 2002.
52. Levenberg P, Elster A. *Guidelines for Adolescent Prevetive Services (GAPS): Clinical Evaluation and Management Handbook*. Chicago: American Medical Association; 1995.
53. Knight JR. Substance abuse in adolescents. In: Parker S, Zuckerman B, Augustyn A, eds. *Developmental and Behavioral Pediatrics: A Handbook for Primary Care, second edition*. New York: Lippincott Williams & Wilkins; 2005.
54. Dhalla S, Zumbo BD, Poole G. A review of the psychometric properties of the CRAFFT instrument. *Curr Drug Abuse Rev.* 2011;4(1):57-64.
55. Winters KC, Kaminer Y. Screening and assessing adolescent substance use disorders in clinical populations. *J Am Acad Child Adolesc Psychiatry.* 2008;47(7):740-744.
56. Csemy L, Knight JR, Starostova O, Sherritt L, Kabicek P, Van Hook S. Adolescents substance abuse screening program: experience with Czech adaptation of the CRAFFT screening test. *Vox Pediatriae.* 2008;8(6):35-36.
57. Lenhart A, Purcell K, Smith A, Zickuhr K. *Social media & mobile internet use among teens and young adults (available on-line: http://pewinternet.org/Reports/Social-Media-and-Young-Adults.aspx).* Washington, D.C.2010.
58. Rojas NL, Sherrit L, Harris S, Knight JR. The role of parental consent in adolescent substance use research. *J Adolesc Health.* Feb 2008;42(2):192-197.
59. Center for Adolescent Substance Abuse Research CsHB. Example Human Subjects Research Protocol 2011; http://www.ceasar.org/isbirt/materials/Example_Human_Subjects_Protocol.doc.
60. Sobell LC, Sobell MB. Timeline followback: a technique for assessing self-reported alcohol consumption. In: Litten RZ, Allen JP, eds. *Measuring alcohol consumption: psychosocial and biochemical methods.* Totowa, NJ: Humana; 1992:41-72.
61. Sobell L, Sobell M. *Alcohol Timeline Followback User's Manual.* Toronto: Addiction Research Foundation;1995.

62. Sobell LC, Brown J, Leo GIS, M.B. The reliability of the Alcohol Timeline Followback when administered by telephone and by computer. *Drug and Alcohol Dependence.* 1996;42:49-54.
63. Lewis-Esquerre JM, Colby SM, Tevyaw TO, Eaton CA, Kahler CW, Monti PM. Validation of the timeline follow-back in the assessment of adolescent smoking. *Drug and Alcohol Dependence.* 2005;79:33-43.
64. Hjorthoj CR, Hjorthoj AR, Nordentoft M. Validity of Timeline Follow-Back for self-reported use of cannabis and other illicit substances - systematic review and meta-analysis. *Addict Behav.* 2011;In press.
65. Saunders JB, Aasland OG, Babor TF, de la Fuente JR, Grant M. Development of the Alcohol Use Disorders Identification Test (AUDIT): WHO Collaborative Project on Early Detection of Persons with Harmful Alcohol Consumption--II. *Addiction.* 1993;88(6):791-804.
66. Babor TF, Higgins-Biddle JC, Saunders JB, Monteiro MG. *AUDIT: The Alcohol Use Disorders Identification Test: Guidelines for use in primary health care. Second Edition.* Geneva, Switzerland: World Health Organization, Department of Mental Health and Substance Dependence;2001.
67. Bohn MJ, Babor TF, Kranzler HR. The Alcohol Use Disorders Identification Test (AUDIT): validation of a screening instrument for use in medical settings. *J Stud Alcohol.* 1995;56(4):423-432.
68. Chan-Pensley E. Alcohol-Use Disorders Identification Test: a comparison between paper and pencil and computerized versions. *Alcohol Alcohol.* Nov-Dec 1999;34(6):882-885.
69. Chung T, Colby SM, Barnett NP, Rohsenow DJ, Spirito A, Monti PM. Screening adolescents for problem drinking: performance of brief screens against DSM-IV alcohol diagnoses. *J Stud Alcohol.* 2000;61(4):579-587.
70. Reinert D, Allen J. The Alcohol Use Disorders Identification Test (AUDIT): A review of recent research. *Alcoholism Clin Exp Research.* 2002;26(2):272-279.
71. Shaffer D, Fisher P, Lucas CP, Dulcan MK, Schwab-Stone ME. NIMH Diagnostic Interview Schedule for Children Version IV (NIMH DISC-IV): description, differences from previous versions, and reliability of some common diagnoses. *J Am Acad Child Adolesc Psychiatry.* 2000;39(1):28-38.
72. Schwab-Stone ME, Shaffer D, Dulcan MK, et al. Criterion validity of the NIMH Diagnostic Interview Schedule for Children Version 2.3 (DISC 2.3). *J Am Acad Child Adolesc Psychiatry.* 1996;35:878-888.
73. Difranza JR, Sweet M, Savageau JA, Ursprung WW. The assessment of tobacco dependence in young users of smokeless tobacco. *Tob Control.* 2011;In press.
74. O'Loughlin J, Difranza J, Tyndale RF, et al. Nicotine-dependence symptoms are associated with smoking frequency in adolescents. *Am J Prev Med.* 2003;25(3):219-225.
75. O'Loughlin J, Tarasuk J, Difranza J, Paradis G. Reliability of selected measures of nicotine dependence among adolescents. *Ann Epidemiol.* 2002;12(5):353-362.
76. Wheeler KC, Fletcher KE, Wellman RJ, Difranza JR. Screening adolescents for nicotine dependence: the Hooked On Nicotine Checklist. *J Adolesc Health.* Sep 2004;35(3):225-230.
77. Martino S, Grilo CM, Fehon DC. Development of the Drug Abuse Screening Test for Adolescents (DAST-A). *Addict Behav.* 2000;25(1):57-70.
78. Yudko E, Lozhkina O, Fouts A. A comprehensive review of the psychometric properties of the Drug Abuse Screening Test. *J Subst Abuse Treat.* 2007;32(2):189-198.
79. Lucas CP, Zhang H, Fisher PW, et al. The DISC Predictive Scales (DPS): Efficiently screening for diagnoses. *J Am Acad Child Adolesc Psychiatry.* 2001;40(4):443-449.
80. Aquilino WS. Interview mode effects in surveys of drug and alcohol use: a field experiment. *Public Opin Q.* 1994;58:210-240.
81. Currivan D, Nyman A, L., Turner CF, Biener L. Does telephone audio computer-assisted self-interviewing improve the accuracy of prevalence estimates of youth smoking? Evidence from the UMass Tobacco Study. *Public Opin Q.* 2004;68(4):542-564.

82. Fowler FJ, Stringfellow VL. Learning from experience: estimating teen use of alcohol, cigarettes, and marijuana from three survey protocols. *Journal of Drug Issues.* 2001;31:643-664.

83. Gfroerer JD, Hughes AL. Collecting data on illicit drug use by phone. In: Turner Cf, Lessler JT, Gfroerer JD, eds. *Survey Measurement of Drug Use: Methodological Issues.* Vol DHHS Publication Number 92_1929. Washington, D.C.: Government Printing Office; 1992:277-295.

84. Babor TF, Kranzler HR, Lauerman RJ. Early detection of harmful alcohol consumption: comparison of clinical, laboratory, and self-report screening procedures. *Addict Behav.* 1989;14(2):139-157.

85. Winters KC, Stinchfield RD, Henly GA, Schwartz RH. Validity of adolescent self-report of alcohol and other drug involvement. *Int J Addict.* 1990;25(11A):1379-1395.

86. Shrout PE, Fleiss JL. Intraclass correlations: uses in assessing rater reliability. *Psychological Bulletin.* 1979;86(2):420-428.

87. National Institute on Alcohol Abuse and Alcoholism. *Alcohol Screening and Brief Intervention for Youth: A Practitioner's Guide*. Washington, D.C.: National Institutes of Health, Department of Health and Human Services; 2011.

88. Cohen J. A coefficient of agreement for nominal scales. *Educational and Psychological Measurement.* 1960;20:37-46.

89. Cohen J. Weighted kappa: nominal scale agreement with provision for scaled disagreement or partial credit. *Psychological Bulletin.* 1968;70(4):213-220.

90. Donovan JE. Estimated blood alcohol concentrations for child and adolescent drinking and their implications for screening instruments. *Pediatrics.* 2009;123(6):e978-981.

91. Carpenter J, Bithell J. Bootstrap confidence intervals: when, which, what? A practical guide for medical statisticians. *Stat Med.* 2000;19(9):1141-1164.

92. Platt RW, Hanley JA, Yang H. Bootstrap confidence intervals for the sensitivity of a quantitative diagnostic test. *Stat Med.* 2000;19(3):313-322.

93. Desu MM, Raghavarao D. Sample size methodology. In: Mellor B, ed. *Statistics in Medicine.* Vol 11. Boston: Academic Press; 1990:562-563.
