# Supplementary material for: Effect of Computer-Based Substance Use Screening and Brief Behavioral Counseling vs Usual Care for Youths in Pediatric Primary Care: A Pilot Randomized Clinical Trial
Source: JAMA Netw Open. 2019 Jun 21;2(6):e196258. doi: 10.1001/jamanetworkopen.2019.6258 (PMC6593643; doi:10.1001/jamanetworkopen.2019.6258)
Supplement: Supplement 2. — eTable 1. Baseline Characteristics of Overall Group and by Study Arm Among Youths Reporting No Use of Alcohol or Other Drugs in the Past 12 Months at Baseline eTable 2. Youths’ Reports of Clinician Counseling and Ratings of Their Visit Among Those With No Reported Use of Alcohol or Other Drugs in the Past 12 Months at Baseline eFigure. Kaplan-Meier Survival Curves for Time to First Use During Follow-Up Among Participants Who Reported No Use of Alcohol or Other Drugs in the Past 12 Months at Baseline [file jamanetwopen-2-e196258-s002.pdf]

Supplementary Online Content

Knight JR, Sherritt L, Gibson EB, et al. Effect of computer-based substance use screening and brief behavioral counseling vs usual care for youths in pediatric primary care: a pilot randomized clinical trial. *JAMA Netw Open*. 2019;2(6):e196258. doi:10.1001/jamanetworkopen.2019.6258

eTable 1. Baseline Characteristics of Overall Group and by Study Arm Among Youths Reporting No Use of Alcohol or Other Drugs in the Past 12 Months at Baseline

eTable 2. Youths' Reports of Clinician Counseling and Ratings of Their Visit Among Those With No Reported Use of Alcohol or Other Drugs in the Past 12 Months at Baseline

eFigure. Kaplan-Meier Survival Curves for Time to First Use During Follow-Up Among Participants Who Reported No Use of Alcohol or Other Drugs in the Past 12 Months at Baseline

This supplementary material has been provided by the authors to give readers additional information about their work.

© 2019 Knight JR et al. *JAMA Network Open.*

eTable 1. Baseline Characteristics of Overall Group and by Study Arm Among Youths Reporting No Use of Alcohol or Other Drugs in the Past 12 Months at Baseline

Baseline characteristic	ALL (*N* = 658[a]) n (%)	cSBI (*n* = 478[a]) n (%)	UC (*n* = 180[a]) n (%)	Test statistic[b]	p-value
Age in years (mean ±SD)	14.3 ± 1.8	14.1 ± 1.8	14.5 ± 1.7	2.63	p<.01
9th-12th graders	296 (49.1)	197 (41.2)	100 (55.2)	.13	p<.01
Girls	326 (49.6)	236 (49.4)	91 (50.3)	.00	p=.84
Race/Hispanic ethnicity				.04	p=.60
White non-Hispanic	282 (42.9)	203 (42.5)	79 (43.9)		
Hispanic	201 (30.5)	143 (29.9)	58 (32.2)		
Other/Multi-race	175 (26.6)	132 (27.6)	43 (23.9)		
Two parents at home	523 (80.0)	380 (79.6)	143 (79.4)	.01	p=.84
College-graduate parent(s)	414 (71.5)	306 (72.2)	108 (69.7)	.02	p=.56
Saw pediatrician at visit	564 (85.7)	412 (86.2)	152 (84.4)	.02	p=.57
Saw a female clinician	438 (66.6)	321 (67.2)	117 (65.0)	.02	p=.60
Had ≥6 prior visits with clinician	390 (59.6)	284 (59.8)	106 (59.2)	.00	p=.89
Rode with driver who had been using alcohol or drugs	43 (6.5)	29 (6.1)	14 (7.7)	.03	p=.44
Hangs out with any friends that use alcohol or drugs	251 (38.1)	169 (35.4)	82 (45.3)	.09	p=.02
Substance-involved siblings[c]	50 (8.9)	40 (9.7)	10 (6.6)	.05	p=.25
Substance-involved parents[c]	35 (5.3)	27 (5.7)	8 (4.4)	.03	p=.53

Abbreviations: UC = cSBI = computer-facilitated Screening and Brief Intervention; Usual Care group; SD = standard deviation; CRAFFT = mnemonic for Car, Relax, Alone, Forget, Family/Friends, Trouble.

[a] Sample denominators for individual variables do not always equal the total group size due to missing responses; valid percentages are reported.

[b] Test statistics for continuous variables are from independent samples t-tests (age); for categorical variables, we present the Cramér's V and associated p-value, a measure of strength of association (similar to a correlation coefficient) among categorical variables.

[e] Percent reporting any "agree" response to scale items from the Personal Experience Inventory assessing substance-involvement of siblings or parents.

© 2019 Knight JR et al. *JAMA Network Open.*

eTable 2. Youths' Reports of Clinician Counseling and Ratings of Their Visit Among Those With No Reported Use of Alcohol or Other Drugs in the Past 12 Months at Baseline

Baseline post-visit assessment measure	Analysis n	cSBI n (%) (n=478[a])	UC n (%) (n=181[a])	ARRR[a,b] (95%CI)
Advised about alcohol[c]	654	433 (91.0)	126 (70.8)	1.30*** (1.17, 1.43)
Advised about cannabis[c]	655	430 (90.3)	127 (70.9)	1.30*** (1.17, 1.44)
Advised about not riding with impaired driver	654	387 (81.5)	79 (44.1)	1.89*** (1.59, 2.25)
Advised about not driving after using substances[d]	296	160 (81.2)	42 (42.4)	1.91*** (1.51, 2.39)
Received information on health and safety risks of…				
Alcohol	653	407 (85.9)	112 (62.6)	1.38*** (1.22, 1.56)
Cannabis	654	396 (83.4)	103 (57.5)	1.47*** (1.28, 1.68)
"Excellent"/ "Very Good" rating of clinician advice[e]	568	348 (79.8)	92 (69.7)	1.13* (1.00, 1.27)
"Very much" likely to follow clinician advice[e]	574	327 (74.3)	85 (63.4)	1.15* (1.00, 1.31)
"Very much" or "Quite a bit" satisfied with visit	653	456 (96.0)	169 (94.9)	1.02 (0.97, 1.06)
Received Contract for Life[f]	254	141 (79.2)	6 (7.9)	11.59*** (5.22, 25.74)

Abbreviations: cSBI = computer-facilitated Screening and Brief Intervention; UC = Usual Care; ARRR = Adjusted Relative Risk Ratio; CI = Confidence Interval.

* p<.05, ** p < 0.01

[a] Adjusted relative risk ratio with UC as the reference group.

[b] Logistic regression with generalized estimating equations (GEE) adjusted for grade group (middle vs. high school), conducted using SUDAAN® v. 11.0.3 software to account for clinician-sampling design.

[c] The "Advised" variables include advice to *not start* alcohol or cannabis/drug use for adolescents who had no prior use of the substance and advice to *stop* further use for those who had used the substance in the past 12 months.

[d] This question was asked of high school students only; analysis denominator for UC=99, cSBI=197.

[e] Among adolescents reporting receiving advice about alcohol or cannabis/drug use; analysis denominator for UC=132, cSBI=436.

[f] This question was added nine months after starting recruitment so analysis includes only those who received this question; analysis denominator for UC=76, cSBI=178.

© 2019 Knight JR et al. *JAMA Network Open.*

eFigure. Kaplan-Meier Survival Curves for Time to First Use During Follow-Up Among Participants Who Reported No Use of Alcohol or Other Drugs in the Past 12 Months at Baseline

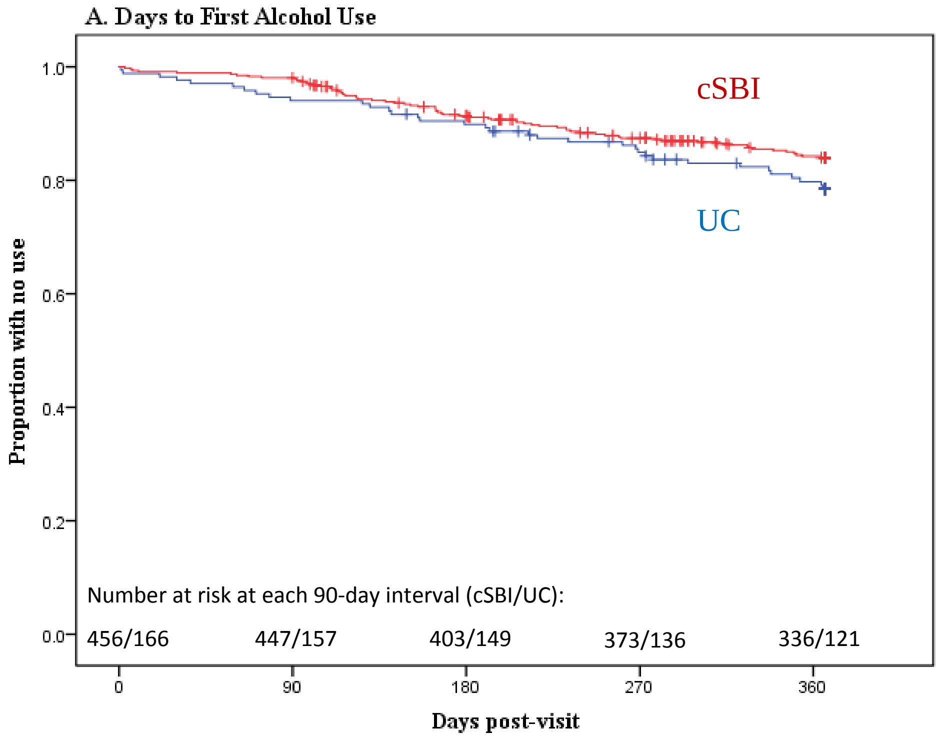

© 2019 Knight JR et al. *JAMA Network Open.*

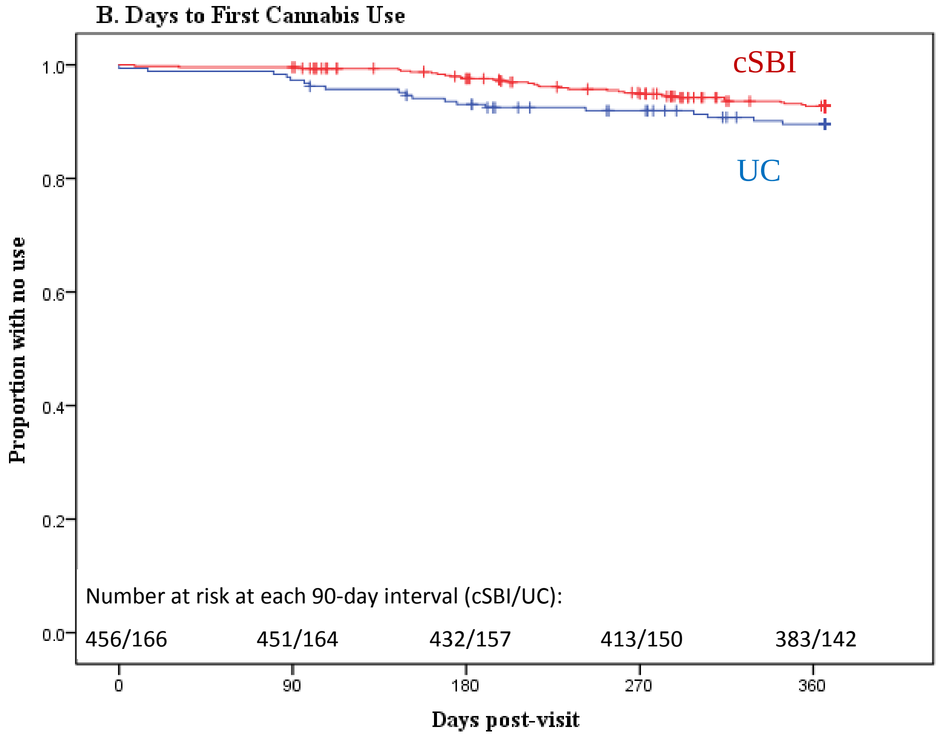

The x-axis displays the number of days during the 12-months follow-up. The red line is the survival curve showing the proportion of adolescents in the computer-facilitated Screening and Brief Intervention (cSBI) group with no use since baseline. The blue line is the curve for those receiving Usual Care (UC). The "+" shows censored observations within each study arm. Above the x-axis, we present the number of adolescents in each arm still at risk for use at each 90-day interval. Medians (interquartile range [IQR]) for number of days to first use by group were the following: for any alcohol use, cSBI= 366 (338-366) and UC=366 (334-366); for cannabis use, cSBI=366 (366-366) and UC=366 (366-366).

© 2019 Knight JR et al. *JAMA Network Open.*
