# Supplementary material for: Effect of Computer-Based Substance Use Screening and Brief Behavioral Counseling vs Usual Care for Youths in Pediatric Primary Care: A Pilot Randomized Clinical Trial
Source: JAMA Netw Open. 2019 Jun 21;2(6):e196258. doi: 10.1001/jamanetworkopen.2019.6258 (PMC6593643; doi:10.1001/jamanetworkopen.2019.6258)
Supplement: Supplement 3. — Data Sharing Statement [file jamanetwopen-2-e196258-s003.pdf]

Data Sharing Statement

Knight. Effect of Computer-Based Substance Use Screening and Brief Behavioral Counseling vs Usual Care for Youths in Pediatric Primary Care. *JAMA Netw Open*. Published June 21, 2019. 10.1001/jamanetworkopen.2019.6258

Data
Data available: Yes
Data types: Deidentified participant data, Data dictionary
How to access data: Please email Dr. Sion Harris at sion.harris@childrens.harvard.edu and cc: erin.gibson@childrens.harvard.edu
When available: beginning date: 12-31-2019

Supporting Documents
Document types: None

Additional Information
Who can access the data: Researchers who proposed use of the data has been approved
Types of analyses: Any purpose
Mechanisms of data availability: Data will be made available after approval of a proposal.
